# Elucidating the involvement of apoptosis in postmortem proteolysis in porcine muscles from two production cycles using metabolomics approach

**DOI:** 10.1038/s41598-021-82929-3

**Published:** 2021-02-10

**Authors:** Danyi Ma, Dong Ho Suh, Jiaying Zhang, Yufan Chao, Alan W. Duttlinger, Jay S. Johnson, Choong Hwan Lee, Yuan H. Brad Kim

**Affiliations:** 1 Department of Animal Sciences, Purdue University, West Lafayette, IN 47907 USA; 2 Department of Bioscience and Biotechnology, Konkuk University, Seoul, 05029 South Korea; 3 USDA-ARS Livestock Behavior Research Unit, West Lafayette, IN 47907 USA; 4 Research Institute for Bioactive-Metabolome Network, Konkuk University, Seoul, 05029 South Korea

**Keywords:** Biochemistry, Cell biology, Molecular biology, Systems biology

## Abstract

Apoptosis has been suggested as the first step in the process of conversion of muscle into meat. While a potential role of apoptosis in postmortem proteolysis has been proposed, the underlying mechanisms by which metabolome changes in muscles would influence apoptotic and proteolytic process, leading to meat quality variation, has not been determined. Here, apoptotic and proteolytic attributes and metabolomics profiling of longissimus dorsi (LD) and psoas major (PM) muscles in pigs from two different production cycles (July–Jan vs. Apr–Sep) were evaluated. PM showed higher mitochondrial membrane permeability (MMP), concurrent with less extent of calpain-1 autolysis and troponin T degradation and higher abundance of HSP27 and αβ-crystallin compared to LD (P < 0.05). Apr–Sep muscles showed concurrence of extended apoptosis (indicated by higher MMP), calpain-1 autolysis and troponin T degradation, regardless of muscle effects (P < 0.05). Metabolomics profiling showed Apr–Sep muscles to increase in oxidative stress-related macronutrients, including 6-carbon sugars, some branched-chain AA, and free fatty acids. Antioxidant AA (His and Asp) and ascorbic acid were higher in July–Jan (P < 0.05). The results of the present study suggest that early postmortem apoptosis might be positively associated with pro-oxidant macronutrients and negatively associated with antioxidant metabolites, consequently affecting meat quality attributes in a muscle-specific manner.

## Introduction

Postmortem proteolysis plays important role in muscle structure fragmentation affecting tenderness and/or water-holding capacity^1,2^. Proteolysis, however, can only explain limited portion of quality variations in pork^3^. Given the exact mechanisms by which postmortem aging governs palatability development are still not fully understood, regulatory factors of postmortem proteolysis require further exploration. Apoptosis, or programmed cell death, refers to a finely regulated and controlled process by which cells recruit innate enzyme systems to break down functional and/or structural compartment to terminate cellular life cycle^4^. Apoptosis was recently proposed as the very first phase in muscle-meat conversion process, and probably has active interaction with postmortem proteolysis^4–6^. Therefore, apoptotic impact on development of organoleptic and/or perceptional quality attributes has become a growing research interest. Notably, damage of mitochondria normal function triggers the release of pro-apoptotic factors^7,8^. As mitochondria are the central organelles for energy metabolism and redox regulation, it is reasonable to postulate that metabolism background of skeletal muscles could evolve in antemortal apoptosis response.

It is well acknowledged that muscle fiber types, which range from glycolytic fast twitch (Type IIB) to oxidative slow twitch (Type I), may be key contributors to meat quality development^9^. Oxidative muscles were reported to have inferior tenderization potential, accompanied with decreased protease activity^10^. As such, postmortem metabolism features need to be profiled in different muscle types to establish linkage between postmortem metabolism, apoptosis, and subsequent proteolysis to shed lights on updated views of muscle to meat conversion. Furthermore, in our recent study, the production batch effect in animal growth and productivity and meat quality attributes were found^11^. Accordingly, one batch of pigs that were weaned in July 2016 and harvested in January 2017 (July–Jan) exhibited inferior productivity shown as decreased hot carcass weight and body fat, compared to another batch of pigs that were weaned in April 2017 and harvested in September 2017 (Apr–Sep)^11^. Moreover, *longissimus dorsi* (LD) and *psoas major* (PM) muscles in the July–Jan group had higher Warner–Bratzler shear force, thaw-purge loss, and cook loss compared to the Apr–Sep group^11^. These findings postulate that discrepancies in live-animal handling by different production environment/season may lead to overall shifted whole-body metabolism, appeared as altered animal growth. Differently regulated metabolism may lead to distinct mitochondria activities and antemortal apoptotic responses, causing inconsistency in meat quality development.

Previous studies have shown the potential of metabolomics technology to elucidate molecular mechanism of meat quality development under various conditions^12,13^. In this regard, high-throughput metabolomics platform and various bioinformatics tools could be applied to identify metabolism fingerprints that were related to apoptosis, and establish linkages between postmortem energy metabolism, apoptosis, and proteolysis in a muscle-meat conversion prospective. Therefore, the objective of the current study was to evaluate apoptotic and proteolytic characteristics and metabolome changes of porcine muscles from two production replicates. We hypothesize that metabolism features can be altered due to muscle type difference or live-animal production batch effects, which could have further impacts on postmortem apoptotic and proteolytic process, leading to meat quality variation. Two porcine muscles (LD and PM) were selected, because they were known to have distinct differences in fiber composition and metabolic features. This study is further elaboration of our recent study^11^, where impacts of muscle type and production batch effect on animal growth and productivity, carcass characteristics, and pork quality attributes were determined in the same porcine muscle samples.

## Results

### Calpain-1 and myofibrillar protein degradation

**Figure 1:**
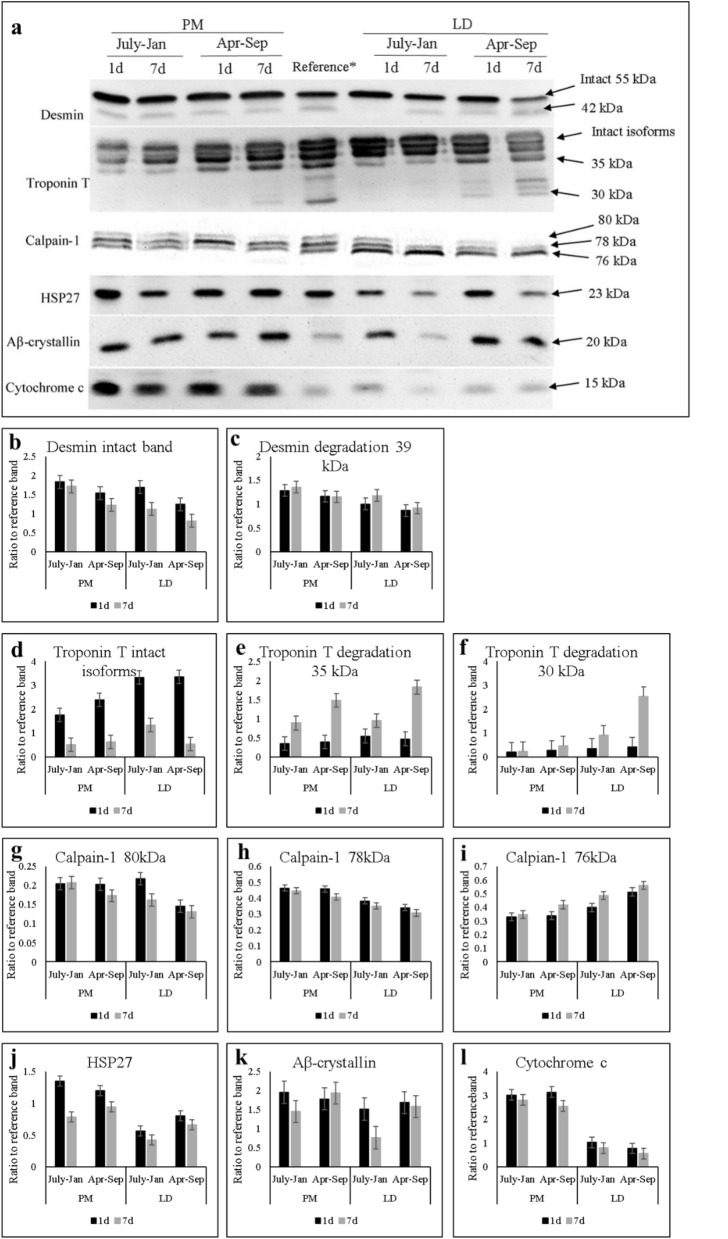
**(a)** A representative set of western blot images showing 1 day vs. 7 days postmortem change of desmin, troponin T, calpain-1, HSP27, αβ-crystallin and cytochrome c of porcine LD (*longissimus dorsi*) and PM (*psoas major*) muscles with two production replicates. Reference: consistently use a 45 min postmortem LD for calpain-1 and a 7 days postmortem LD for desmin, troponin T, HSP27, αβ-crystallin and cytochrome c. July–Jan: Pigs weaned in July 2016 and harvested in January 2017; Apr–Sep: Pigs weaned in April 2017 and harvested in September 2017. All western blots were performed in separate gels in independent manners (please see supplementary information for the entire western blot images). **(b–l)** Densitometry analysis of each protein bands.

The extent of calpain-1 autolysis by western blot analysis was determined as quantifying three bands (80, 78, and 76 kDa; Fig. 1). Overall, no significant interactions among production replicate, muscle or aging effects were observed on calpain-1 autolysis. Regardless of replicate or muscle effects, intact calpain-1 (80 kDa) and partial autolyzed 78 kDa subunits were decreased (*P* < 0.05, Table 1), while its 76 kDa subunits increased in abundance (*P* < 0.005; Table 1). Particularly, production replicate effect affected protein abundance of all three bands (Table 1), where Apr–Sep exhibited increased 76 kDa subunit (*P* = 0.001) and decreased 78 kDa (*P* = 0.054) and 80 kDa subunits (*P* = 0.002) compared to July–Jan counterparts. Within both aging time point (1 day vs. 7 days postmortem), the LD from both Apr–Sep and July–Jan were found to have lower abundance of un-autolysed calpain-1 80 kDa bands (*P* < 0.01) and partial autolyzed 78 kDa bands (*P* < 0.001) and higher abundance of fully autolyzed 76 kDa product (*P* < 0.0001) than the PM muscles of the same production replicate (Table 1).

**Table 1:** Effect of different production replicates and muscle types on proteolysis and apoptosis features of porcine skeletal muscles over 7-day postmortem aging.

Parameters^a,b,c^	Replicate (R)^d^		Muscle (M)^e^		Aging (A)		S.E.M^f^	P-value		
	Apr–Sep	July–Jan	LD	PM	1 day	7 days		R	M	A
MMP	0.082	0.092	0.092	0.081	0.094	0.079	0.002	< .001	< .001	< .0001
Cytochrom c	1.76	1.91	0.79	2.88	1.99	1.68	0.22	0.20	< .0001	0.003
HSP27	0.90	0.78	0.61	1.07	0.98	0.70	0.08	0.018	< .0001	< .0001
Αβ-crystallin	1.75	1.42	1.39	1.78	1.74	1.43	0.30	0.071	0.029	0.092
Calpain-1 80 kDa	0.16	0.20	0.16	0.20	0.19	0.17	0.02	0.002	0.004	0.025
Calpain-1 78 kDa	0.38	0.41	0.35	0.44	0.41	0.38	0.02	0.054	< .0001	0.050
Calpain-1 76 kDa	0.46	0.39	0.49	0.36	0.40	0.45	0.03	0.001	< .0001	0.005
Troponin T intact	1.72	1.73	2.14	1.32	2.71	0.75	0.48	0.98	0.071	0.002
Troponin T Degradation 35 kDa	1.04	0.68	0.95	0.78	0.44	1.29	0.19	0.038	0.17	< .001
Troponin T Degradation 30 kDa	0.92	0.43	1.05	0.30	0.32	1.03	0.40	0.10	0.039	0.037
Desmin intact	1.21	1.59	1.22	1.58	1.58	1.22	0.17	0.002	0.094	< .001
Desmin degradation 39 kDa	1.02	1.21	0.99	1.24	1.08	1.15	0.12	0.085	0.046	0.22

^a^MMP (mitochondrial membrane permeability) was obtained by measuring UV absorption peak at 540 nm of the isolated mitochondria suspensions, higher number indicates lower MMP.

^b^Protein abundance except calpain-1 was expressed as relative ratio of band intensity compared to the corresponding bands of the reference samples.

^c^Three bands of 80, 78, and 76 kDa of calpain-1 were quantified and expressed as the ratio of each band that relative to the total intensity.

^d^Replicate: July–Jan, pigs weaned in July 2016 and harvested in January 2017; Apr–Sep: pigs weaned in April 2017 and harvested in September 2017.

^e^Muscle: LD, longissimus dorsi; PM, psoas major.

^f^Pooled standard errors of means.

**Figure 2:**
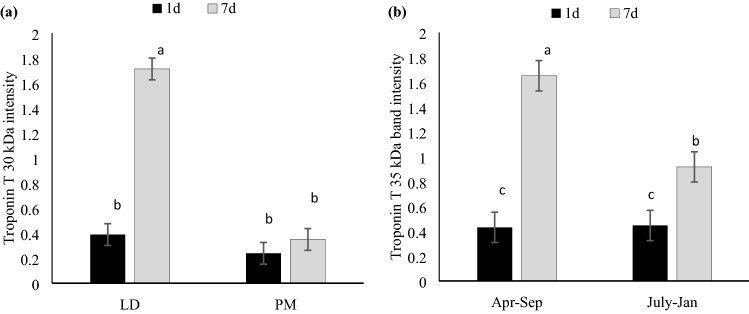
Densitometric analysis of troponin T degradation of porcine LD (longissimus dorsi) and PM (psoas major) muscles with two production replicates. A. Muscle by aging interaction (P = 0.02) on troponin T degradation product intensity at 30 kDa; B. Replicate by aging interaction tendency (P = 0.054) on troponin T degradation product intensity at 35 kDa; Results were displayed as means ± standard error. **(a–c)** Means with different number are significantly different (P < 0.05). July–Jan: Pigs weaned in July 2016 and harvested in January 2017; Apr–Sep: Pigs weaned in April 2017 and harvested in September 2017.

The abundance of intact and degradation products of two myofibrillar proteins, desmin and troponin T, were analyzed (Fig. 1 and Table 1). No significant three-way interactions were found. Intact product of desmin was significantly affected by production replicates and aging times, but not muscle type. Intact desmin was decreased over aging (*P* < 0.001), which was found in overall greater abundance in July–Jan weaned pigs compared with Apr–Sep replicates (*P* = 0.002; Table 1), regardless of aging time or muscle type. Conversely, muscle type effect was observed in desmin degradation, where LD muscles showed less desmin degradation products compared to the PM muscles (*P* = 0.046). Intact product of troponin T were decreased over aging (*P* < 0.01). While troponin T intact product was not responsive to any of the treatment factors, degradation products, which were quantified at 35 kDa and 30 kDa, were decreased with aging (P < 0.05, Table 1). The 35 kDa bands were affected by the replicate × aging (R × A) interaction: both replicates showed similar levels at 1 day postmortem aging, however, Apr–Sep showed further extended degradation at 7 days postmortem compared to July–Jan samples (Fig. 2, *P* < 0.05). Moreover, a muscle × aging interaction tended to affect troponin T degradation product at 30 kDa, where LD showed markedly higher troponin T degradation product than PM at 7 days aging (Fig. 2, *P* < 0.05).

### Mitochondria membrane permeability (MMP) and cytochrome c

MMP were determined to evaluate the extent of structural integrity loss of mitochondria membranes in LD and PM samples. Significant production replicate and muscle type effects on MMP were found (*P* < 0.0001, Table 1). Apr–Sep muscles showed increased MMP compared to July–Jan replicate, suggested higher mitochondrial membrane integrity in July–Jan compared to Apr–Sep (*P* < 0.001). In terms of muscle effect, LD had lower MMP than PM, meaning more integrated mitochondrial membrane compared to PM counterpart (*P* < 0.001).

Western blots were performed to evaluate cytochrome c abundance in LD and PM samples from different production replicates and aging times. PM samples had higher cytochrome c abundance compared to LD (*P* < 0.0001, Table 1). However, no significant difference in cytochrome c abundance was found within production replicates (*P* > 0.05; Table 1).

### Small heat shock proteins

**Figure 3:**
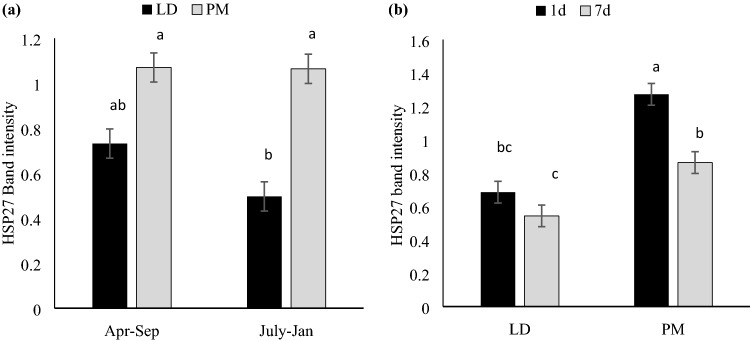
Densitometric analysis of HSP27 of porcine LD (*longissimus dorsi*) and PM (*psoas major*) muscles with two production replicates. **(a)** Muscle by replicate interaction (P = 0.024); **(b)** Muscle by aging interaction (P = 0.009); Results were displayed as means ± standard error. **(a–c)** Means with different number are significantly different (P < 0.05). July–Jan: Pigs weaned in July 2016 and harvested in January 2017; Apr–Sep: Pigs weaned in April 2017 and harvested in September 2017.

Western blots in HSP27 and αβ-crystallin showed single bands without degradation products (Table 1 and Fig. 1). Neither aging time nor production replicate influenced αβ-crystallin abundance (Table 1). However, there was a significant muscle effect, where abundance of αβ-crystallin was higher in PM than LD (*P* = 0.029, Table 1). HSP27 abundance, on the other hand, was affected by replicate × muscle (R × A) and muscle × aging (M × A) interactions (Fig. 3): LD had decreased HSP27 in July–Jan group, but PM did not change over the two replications; also, HSP27 decreased over aging in both LD and PM, while PM had further degradation compared to LD. Even though PM had further degradation, the overall abundance was higher in PM, regardless of aging time, which was corroborated with αβ-crystallin.

### Metabolomics analysis

**Figure 4:**
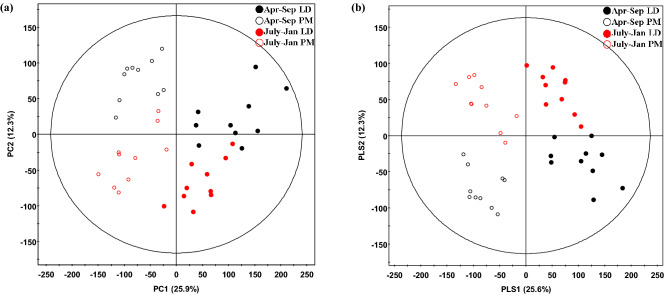
PCA and PLS-DA score plots of metabolome profiling porcine LD (*longissimus dorsi*) and PM (*psoas major*) muscles from two production replicates. The plots were generated using the SIMCAP-P + software (version 12.0, Umetrics, Umea, Sweden); **(a)** PCA score plot, R^2^X = 0.49, Q^2^ = 0.336; **(b)** PLS-DA score plot, R^2^X = 0.559, R^2^Y = 0.981, Q^2^ = 0.835, P < 0.05. July–Jan: Pigs weaned in July 2016 and harvested in January 2017; Apr–Sep: Pigs weaned in April 2017 and harvested in September 2017.

Metabolomics profiling of LD and PM samples from both production replications were obtained using GC-TOF–MS/MS platform with multivariate statistical analysis. The PCA score plots indicated that all experimental groups were notably different from each other, as it showed separation of LD vs. PM muscles from July–Jan vs. Apr–Sep groups (Fig. 4a). The samples primarily separated according to muscle type along the first principal component (PC1), which explained 25.9% of the total variance of the model. In addition, PC2 could explain 12.3% of the total variance of the data set, which separated July–Jan and Apr–Sep muscle samples (Fig. 4a).

While the PLS-DA score plot showed a pattern similar to PCA score plots, this model was used to identify metabolites that differentially presented between muscle and/or production replicate groups (Fig. 4b, R^2^X = 0.559, R^2^Y = 0.981, Q^2^ = 0.835, *P* < 0.05). Overall, the first and second PLS component (PLS1 and PLS2) accounted for 25.6% and 12.3% of the possible variance, respectively (Fig. 4). For the effect of muscle, 64 metabolites were differentially presented in LD vs. PM (VIP1 > 1, from PLS1); for the effect of replicate, 84 metabolites were differentially presented July–Jan vs. Apr–Sep (VIP2 > 1 from PLS2). For all the differentially presented metabolites, major groups can be categorized into carbohydrates, organic acids, free amino acids (AA), fatty acids/lipids, and nucleotide-related metabolites (Table 2). In Apr–Sep, higher abundance of macronutrients was found, including 6-carbon sugars such as glucose and fructose, 14–18 carbon free fatty acids (FFA), and branched-chain amino acids (BCAA) including Leu, Ile, and Val.

**Table 2:** Differentially presented metabolites in LD vs. PM with two production replicates.

RT	Mass	VIP[1] (muscle)	VIP[2] (replicates)	Metabolites^a^	Higher in muscle	Higher in replicate
**Amino acids**						
9.54	156	1.92	1.47	5-Oxoproline	PM	Apr–Sep
8.68	174	0.84	1.68	Alanine	LD	July–Jan
9.08	218	1.31	1.05	Aminomalonic acid	LD	July–Jan
9.1	232	1.32	1.13	Aspartic acid	PM	July–Jan
9.83	329	0.07	1.07	Creatinine		July–Jan
9.56	174	0.25	1.22	GABA		Apr–Sep
10.26	246	1.62	1.45	Glutamic acid	PM	Apr–Sep
7.61	174	1.57	1.41	Glycine	PM	July–Jan
15.97	159	0.6	1.09	Histidine		July–Jan
7.48	158	1.28	1.08	Isoleucine	PM	Apr–Sep
6.06	188	0.23	1.09	Leucine		Apr–Sep
9.48	176	1.48	1.33	Methionine	PM	Apr–Sep
11.75	174	0.42	1.04	Ornithine		July–Jan
10.37	218	1.4	1.13	Phenylalanine	PM	Apr–Sep
7.53	216	1.25	0.94	Proline	PM	July–Jan
8.09	204	1.67	1.25	Serine	PM	Apr–Sep
8.34	219	1.72	1.23	Threonine	PM	July–Jan
6.7	218	1.45	1.14	Valine	PM	Apr–Sep
**Carbohydrates**						
12.28	307	1.99	1.43	Fructose	LD	Apr–Sep
12.21	103	2	1.43	Fructose	LD	Apr–Sep
13.1	333	1.4	1.19	Gluconic acid	LD	Apr–Sep
12.41	160	1.83	1.36	Glucose	LD	Apr–Sep
12.53	160	1.85	1.39	Glucose	LD	Apr–Sep
12.31	160	1.96	1.43	Glucose	LD	Apr–Sep
7.82	189	0.2	1.01	Glyceric acid		Apr–Sep
7.3	205	1.34	1.05	Glycerol	PM	July–Jan
17.1	204	1.31	1.03	Lactose	LD	Apr–Sep
16.91	204	1.32	1.03	Lactose	LD	Apr–Sep
17.41	361	1.38	1.08	Maltose	LD	Apr–Sep
17.27	361	1.44	1.1	Maltose	LD	Apr–Sep
13.66	191	1.97	1.58	Myo-inositol	PM	Apr–Sep
11	246	0.07	1.48	Carbohydrate 1		Apr–Sep
12.9	204	1.64	1.18	Carbohydrate 10	LD	Apr–Sep
13.25	318	2.01	1.51	Carbohydrate 11	PM	Apr–Sep
14.62	315	1.82	1.35	Carbohydrate 12	LD	July–Jan
14.75	204	1.65	1.26	Carbohydrate 13	LD	July–Jan
14.97	315	2	1.44	Carbohydrate 15	LD	Apr–Sep
14.98	387	2.01	1.44	Carbohydrate 16	LD	Apr–Sep
15.05	160	1.98	1.42	Carbohydrate 17	LD	Apr–Sep
15.15	387	1.97	1.41	Carbohydrate 18	LD	Apr–Sep
15.22	204	1.63	1.27	Carbohydrate 19	LD	July–Jan
11.74	204	1.63	1.25	Carbohydrate 2	LD	Apr–Sep
15.34	387	1.85	1.34	Carbohydrate 21	LD	Apr–Sep
11.77	217	1.73	1.24	Carbohydrate 3	LD	July–Jan
11.85	204	1.76	1.32	Carbohydrate 4	LD	Apr–Sep
11.98	217	1.16	1.1	Carbohydrate 5	LD	Apr–Sep
12.34	204	1.68	1.21	Carbohydrate 7	LD	Apr–Sep
12.66	319	1.28	1.25	Carbohydrate 8	PM	Apr–Sep
12.9	217	1.47	1.09	Carbohydrate 9	LD	Apr–Sep
**Lipids**						
15.7	205	0.65	1.12	1-*O*-hexadecylglycerol		Apr–Sep
11.39	357	0.65	1.01	alpha-Glycerophosphoric acid		Apr–Sep
15.12	117	1.49	1.2	Arachidonic acid		Apr–Sep
11.13	243	0.98	1.83	beta-Glycerophosphoric acid		Apr–Sep
17.2	399	0.38	1.18	Glycerol monostearate		
16.07	218	0.66	1.2	Glyceryl 2-palmitate		Apr–Sep
17.02	218	0.16	1.1	Glyceryl 2-stearate		
14.2	337	0.42	1.89	Linoleic acid		Apr–Sep
15.24	343	1.03	0.82	Myristic acid		
15.34	128	0.51	1.15	Oleamide		July–Jan
14.22	199	1.07	1.98	Oleic acid		Apr–Sep
13.17	117	0.54	1.99	Palmitic acid		Apr–Sep
11.54	299	1.52	1.12	Phosphoethanolamine		Apr–Sep
14.35	341	0.85	1.14	Stearic acid		Apr–Sep
**Purine/pyrimidine**						
6.54	166	1.41	1.07	3-Hydroxy-6-methylpyridine	LD	
7.9	241	1.55	1.55	5-Hydroxymethyluracil	PM	Apr–Sep
17.42	236	0.86	1.6	5′-Methylthioadenosine		July–Jan
19.13	169	1.19	1.44	Adenosine 5′-monophosphate	PM	July–Jan
17.3	324	1.66	1.24	Guanosine	PM	July–Jan
11.74	265	1.72	1.37	Hypoxanthine	PM	Apr–Sep
16.32	230	1.44	1.08	Inosine	PM	July–Jan
18.65	169	1.27	1.27	Uridine 5′-monophosphate	PM	July–Jan
**Organic acid**						
6.11	117	1.3	1.07	2-Hydroxybutyric acid	PM	July–Jan
9.68	247	1.67	1.49	2-Hydroxyglutaric acid	PM	Apr–Sep
6.62	131	0.99	1.06	3-Hydroxy-3-methylbutyric acid	PM	Apr–Sep
6.86	233	1.65	1.33	4-Hydroxybutyric acid	PM	Apr–Sep
7.91	245	1.55	1.37	Fumaric acid	PM	Apr–Sep
12.76	361	1.08	0.99	Galactonic acid 1,4-lactone	LD	July–Jan
5.23	177	1.43	1.05	Glycolic acid	PM	
8.88	218	0.86	1.52	Homoserine		July–Jan
9.21	233	1.69	1.65	Malic acid	PM	Apr–Sep
5.36	217	1.32	1.26	Pyruvic acid	PM	Apr–Sep
7.63	247	1.68	1.33	Succinic acid	PM	July–Jan
7.14	189	0.54	1.62	Urea		Apr–Sep
12.7	332	0.27	1.59	Ascorbic acid		July–Jan
**Others**						
11.65	328	0.93	1.11	Ammeline		Apr–Sep
10.23	255	1.18	0.99	Melamine	PM	
10.44	117	0.53	1.18	N.I 5		
12.52	305	1.6	1.25	N.I 6		
12.95	291	1.27	0.94	Pantothenic acid	PM	

^a^Metabolites showing significant differences (VIP > 1 and *P* < 0.05) between muscles and production replicates.

**Figure 5:**
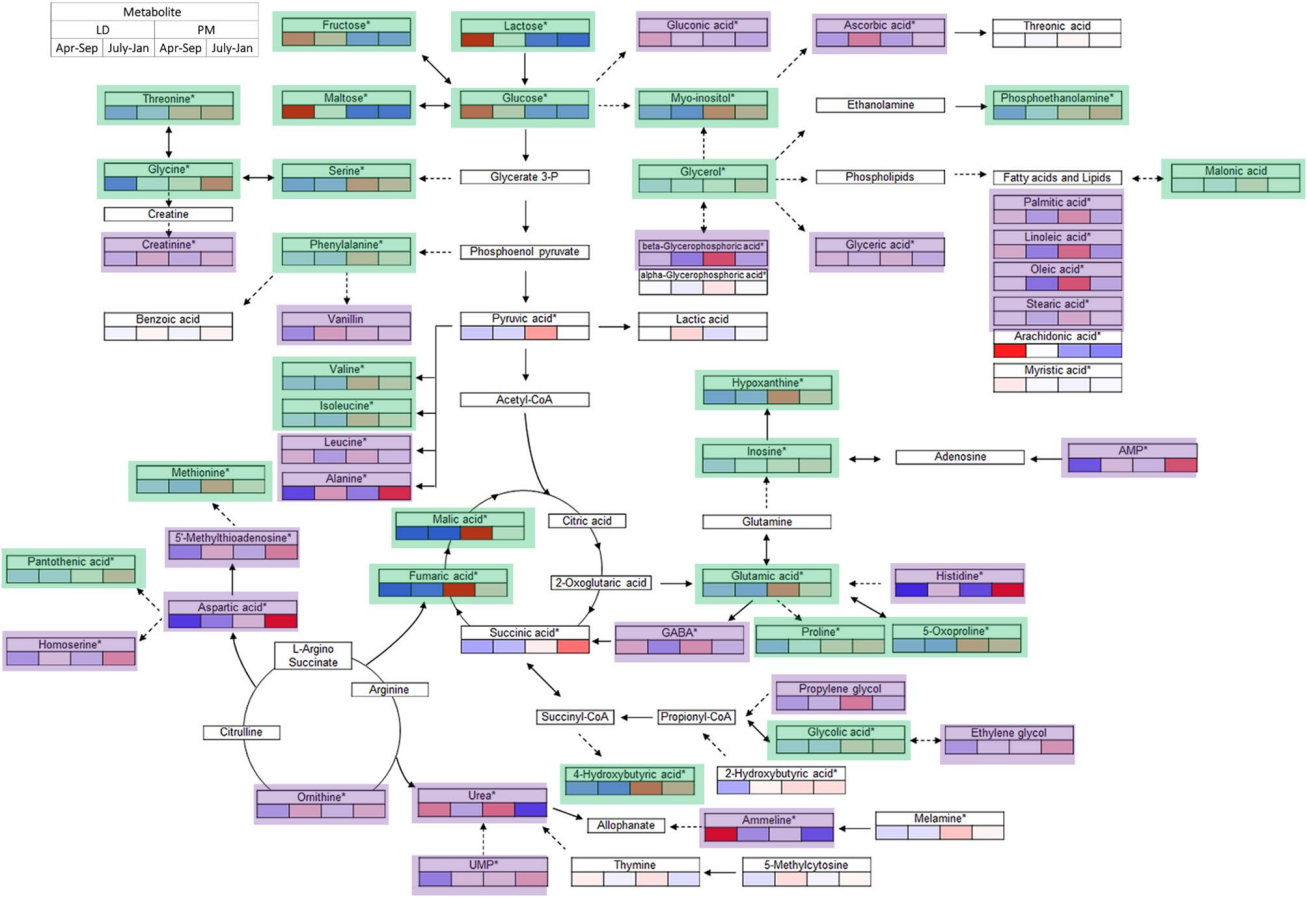
Pathway analysis of differentially affected metabolites and pathways in porcine LD (*longissimus dorsi*) and PM (*psoas major*) muscles from two production replicates. July–Jan: Pigs weaned in July 2016 and harvested in January 2017; Apr–Sep: Pigs weaned in April 2017 and harvested in September 2017.

On the other hand, July–Jan muscles showed elevated antioxidant compounds, such as assortments of redox regulating AA and ascorbic acids (Table 2). Especially, aspartic acid and histidine were higher in both LD and PM of July–Jan, while γ-amino-butyric acid (GABA) and β-glycerophosphoric acid were decreased. Further pathway analysis suggested that Apr–Sep muscles went through further extent of cellular catabolism, including urea cycle and ATP degradation (Fig. 5). These observations corroborated with production replication discrepancies in proteolytic and apoptotic features, with detailed biological interpretations elaborated in the following section.

## Discussion

The idea that apoptosis and proteolysis have coupling activities has been well-supported in various model organisms. Calpain-1, one of the most dominant proteases that retain determinant proteolytic activity during postmortem aging, is among the universal key modulators of apoptosis process^14^. While calpain-1 activation is essential for the subsequent enzymatic activation of the critical apoptotic effector caspase-3^15^, activated caspase-3 cleaves calpastatin, the allosteric inhibitor of calpain-1, leading to further release of calpain-1 enzymatic activity^16^. Moreover, calpain-1 induces endoplasmic reticulum stress, which facilitates cardiac muscle apoptosis under hypoxia conditions^17^. Although direct experimental evidence in postmortem skeletal muscles would need further investigation, degree of calpain-1 autolysis may be related to the onset of apoptotic process to a certain extent. In this regard, we postulate that antemortal apoptosis may pose directly impact on muscle structural protein degradation during postmortem aging, through modulating enzyme activity of calpain-1.

Muscle samples from pig carcasses that were produced in Apr–Sep showed higher levels of MMP, indicating a less integrated membrane system. While higher MMP in Apr–Sep may suggest more extent of apoptosis, Apr–Sep samples also exhibited further calpain-1 autolysis, which was corresponded with increased troponin T and desmin degradation throughout the aging period. The production cycle impact on HSP27 was muscle-type specific, where Apr–Sep samples, which exhibited further degree of proteolysis, showed elevated HSP27 abundance in LD, however, such change was not observed in PM. Overall, extended proteolysis in Apr–Sep muscles was concurrent with further apoptosis, whereas July–Jan showed concurrent decrease in extent of proteolysis and apoptosis features. The current observations suggested a possible role of apoptosis facilitating downstream proteolysis events, subsequently resulting in improved tenderization in Apr–Sep muscles as previously reported in the parallel study^11^.

With regard to muscle type discrepancies in apoptosis and proteolysis, muscle specificity was found in apoptosis-proteolysis crosstalk. While PM muscles showed increased MMP, lower extent of calpain-1 autolysis was found in PM compared to LD. Moreover, PM muscles exhibited higher HSP27 and αβ-crystallin abundance regardless of aging treatment. It should be noted that the previous studies determined that PM had an inferior tenderization potential, while it is considered as tender cut in meat retailing^10,11^. From a stress-defending perspective, PM is red-oxidative, slow twitch muscle, experienced higher level of oxidative stress^18^. Oxidative conditions are known to inhibit calpain-1 activation^19^, whereas cellular oxidative stress level also facilitates onset of apoptosis^20^. This may help to explain the concurrence of extended apoptosis, higher small HSPs and lower calpain-1 autolysis in PM.

Moreover, metabolomics analysis suggested that higher oxidative stress was concurrent with further extent of cell death in Apr–Sep, whereas elevated stress defending response was found in July–Jan, which could be favoring anti-apoptotic activities. The GC-TOF–MS metabolomics showed that in general, Apr–Sep had more abundant macronutrients including simple sugar and a variety of carbohydrate species, BCAA, and 14–18 carbon saturated FFA, whereas July–Jan was characterized with higher level of antioxidant or anti-stress molecules. Fourteen lipids were found to be differentially presented between production replicates, including 6 FFAs, 3 glycolipids, 1 glycerophospholipid, and 1 sterol lipid (Table 2). Particularly, except for oleamide, which was more abundant in July–Jan, all the differentially presented lipid compounds were elevated in Apr–Sep muscles. Exposure of skeletal muscles to lipid surplus was recognized as a trigger of metabolic dysfunction^21^. It was also suggested that saturated FFA are pro-apoptotic compounds to the cells, primarily due to generation of intermediates with mitochondrial toxicity^22,23^. In the current study, 14:0, 16:0, 18:0 and 18:1 were identified with higher abundance in Apr–Sep. Metabolism of excessive FFA in skeletal muscle involves in generation of signaling molecules such as diacylglycerol (DAG) and ceramide, leading to insulin resistance and hence promoting oxidative damage of the tissue and trigger apoptosis^21,24,25^. Therefore, it could be postulated that elevated carbohydrates, BCAA and FFA content in Apr–Sep pigs may be associated with early or extended onset of apoptosis, resulting in improved proteolysis potential of postmortem muscles.

Except for lipids, in the current study, eight carbohydrates were assigned to positive annotations, including fructose, glucose, lactose, maltose, gluconic acid, glyceric acid, glycerol, and myo-inositol. Cell respiration (glycolysis and TCA cycle) intermediates, including pyruvic acid, fumaric acid, malic acid, and succinic acid, were also identified as differentially presented metabolites in between production replicates. Additionally, with regard to nitrogen metabolism, 19 associate metabolites have been found differentially presented in between production replicates, including 13 alpha AA. Since muscle is the largest reservoir of proteins, it plays key role in AA metabolism and protein turn-over^26^. As such, a significant change in AA profile indicated shifted energy metabolism, protein homeostasis, and signaling transduction between the two production replicates.

Urea cycle is closely linked to TCA cycle; the primary modulating hinge includes glutamate^27^. As urea cycle is responsible for ammonia detoxification and nitrogen excretion, elevated urea in Apr–Sep could be an indicator of further AA catabolism, while elevated ornithine and Asp may suggest a more conserved nitrogen utilization in July–Jan pigs, allowing for more amino groups stayed in protein/free AA pool rather than expelled out of the innate environment system (Fig. 4). With increased amino groups disposed, elevated carbon skeleton should be processed via TCA cycle^27^. For example, during catabolism of glutamate family molecules, Gln, Arg, His, Pro, and ornithine are first converted into Glu. Deamination of Glu results in α-ketoglutarate production, which could enter TCA cycle as a key intermediate, favoring respiratory flux and/or gluconeogenesis^27^. Meanwhile, potentially enhanced gluconeogenesis in Apr–Sep, which was suggested by increases in glucose, fructose, and lactose, could in turn favor glycolysis and TCA flux. Under various circumstance, the induction of TCA flux generates oxidative stress and inflammatory response^28,29^. Considering the aforementioned increases of 6-carbon sugars and TCA intermediates in Apr–Sep, these observations together suggested elevated cellular oxidative stress in Apr–Sep porcine muscles, which could be favoring apoptosis settlement.

In the perspective of structural AA profile, Ile, Leu, Val, Met, Phe, Glu, Ser, and 5-oxoproline were more abundant in Apr–Sep, whereas His, Asp, Gly, Pro, Thr, and creatinine, were elevated in July–Jan. It is established that BACC including Ile, Leu, and Val are energy providing AAs as well as major protein-turnover modulators in skeletal muscles^30^. Energy-generating catabolism of BCAA produce reactive oxygen species, free radical molecules that cause oxidative stress, facilitating the onset of cell death process^31,32^. Moreover, Met, Phe and 5-oxoproline are also among the AAs related to potential cellular oxidative stress under both health and pathogenic conditions^33^. Particularly, Met residuals in proteins are susceptible to oxidative attack. Met restriction prevents unwanted shift of AA metabolism, protecting DNA and cell structure integrity^34^. On the other hands, AAs that are related to redox regulation and antioxidant activity, including His^35^, Gly^36^, Thr^37^, Asp^38,39^, Ala^40^, and Pro, were more abundant in July–Jan. Pharmaceutical dose or dietary supplementation of these AAs could alleviate cellular stress under catabolic conditions. In addition, GABA is a well-known inhibitory neuro-transmitter, which was lower in July–Jan. Decreased plasma GABA level is associated with a wide range of mental/physiological distress^41^. Hence, lower GABA level may be an indicator of elevated stress response in July–Jan pigs. In fact, these pigs experienced significantly higher incidence of respiratory infection and antibiotic treatment during growing-finishing phase, indicating a compromised health status in comparison with Apr–Sep replicate^42^. Therefore, it is reasonable to speculate that elevated stress level was occurred in July–Jan, increasing abundance of anti-stress AAs and other antioxidant metabolites. The increased antioxidant metabolites may support anti-apoptotic activity during muscle to meat conversion, results in delayed proteolysis.

Changes of purine/pyrimidine metabolites, consistently, showed that Apr–Sep muscles underwent further degradation of cell structure integrity, in a perspective of adenosine triphosphate (ATP) breakdown. It was generally acknowledged that ATP can be catabolized into series of intermediates, including ADP/AMP, inosine-mono phosphate (IMP), inosine, and eventually hypoxanthine and uric acid^43^. In the current results, July–Jan muscles showed higher abundance of AMP, UMP, inosine, guanosine, and 5′-Methylthioadenosine, which were located in the upstream of ATP catabolism pathway, whereas hypoxanthine and 5-hydroxymethyluracil were more elevated in Apr–Sep, which belong to further degradation product of ATP catabolism. It was well established that oxidative stress favors ATP catabolism, render increased hypoxanthine in bio-system^44^. As such, elevated hypoxanthine level in Apr–Sep was an indicator of further degradation of cell compartments, consistent with extended tendency of apoptosis and proteolysis activities.

To sum up, the present study found that both production replicate batch effect and muscle specificity affected proteolytic changes, small heat shock proteins and apoptosis characteristics in porcine muscles. PM showed advanced MMP decrease during early postmortem, compared to LD, as well as increased HSP27 and αβ-crystallin, less extent of proteolysis, and lower degree of calpain-1 autolysis. Moreover, consistent with replication discrepancies in meat tenderness development^11^, muscles harvested in July–Jan showed lower MMP as an indicator of less extent of apoptosis, concurrent with less extent of calpain-1 autolysis and proteolysis, regardless of muscle effect. Moreover, changes in global metabolomics profiling between production replicates suggested that Apr–Sep muscles retained higher level of oxidative-stress-associated macronutrients, including 6-carbon sugars, 14–18 carbon FFA, and BCAA, therefore possibly making muscle cells more prone to antemortal apoptotic process. On the other hand, July–Jan muscle samples showed increases in stress defending compounds such as His, Asp, Gly, Pro, Thr, ascorbic acid, inosine, and guanosine. Taken together, these chemical finger prints support a postulation that advanced oxidative stress may favor the onset of apoptosis, subsequently resulting in proteolysis and meat tenderization process, whereas higher anti-apoptotic, stress defending metabolites may defer antemortal cell death responses, thus consequently leading to adverse impacts on proteolytic potential of postmortem muscles.

## Methods

### Animals and muscle processing

The detailed information regarding live animal handling and growth performance was reported in previous study^42^. Animal husbandry and experimental procedures were approved by the Purdue University Animal Use and Care Committee (protocol #1603001385). All authors complied with the ARRIVE guidelines. In brief, two repetitions of pigs (barrows and gilts, n = 240 pigs/replicate) were weaned at 19 days and reared for 4 month before marketing, which were replicated during July 2016 to January 2017 (July–Jan) and April 2017 to September 2017 (Apr–Sep). Ten animals (age of 5-month, BW 120.28 ± 1.32 kg) were randomly chosen from each July–Jan replicate and Apr–Sep replicate, and slaughtered in Purdue University Meat Laboratory. During carcass chilling, LD and PM muscles were sampled at both 1 days and 7 days postmortem, snap frozen and stored at − 80 °C.

### SDS-PAGE and western blots

Gel samples were prepared in accordance with Kim, Huff-Lonergan, Sebranek, and Lonergan (2010) with minor modifications^3^. All methods were performed in accordance with the relevant guidelines and regulations**.** One gram of muscle sample was homogenized in 10 mL of extraction buffer (10 mM phosphate, 2% w/v sodium dodecyl sulfate (SDS), pH 7.0 at 20 °C). After centrifugation at 1500×*g* for 15 min at 4 °C, supernatant was diluted to protein concentration of 6.4 mg/mL, and mixed with 0.5 volume of tracking dye buffer (3 mM EDTA, 3% w/v SDS, 20% v/v glycerol, 0.003% w/v bromophenol blue, and 30 mM Tris–HCl; pH 8.0) and 0.1 volume of 2-mercaptoethanol, making the final protein concentration 4.0 mg/mL. The mixtures were incubated in 50 °C heat block for 20 min and stored at − 80 °C.

The following loaded total proteins were applied to each of the targeted proteins: 20 μg for troponin T, 40 μg for desmin, calpain-1, HSP27, αβ-crystallin, and cytochrome c. Gels were electrophoresed on a Hoefer SE260 unit at a constant voltage of 25 v for approximately 12 h in running buffer (3.021% Tris, 14.4% glycine, 2% SDS, 0.058% EDTA). Proteins were then transferred to polyvinylidene fluoride membranes and were blocked for 1 h under 25 °C using PBS-Tween (PBST) solution (pH 7.0) containing 5% nonfat dry milk. Prior to hybridization, membranes were cut according to a non-fluorescent protein ladder (ThermoFisher PI26616). Membranes were probed with primary antibody solutions at 4 °C for 8 h overnight, which were prepared using PBST containing 3% nonfat dry milk. The following antibodies were used: 1:10,000 mouse monoclonal anti-desmin IgG (Sigma-Aldrich D1022), 1:20,000 anti-troponin T IgG (Sigma-Aldrich T6277), 1:10,000 mouse monoclonal anti-Mu-calpain (calpain-1) IgG (ThermoFisher MA3-940), 1:3000 mouse monoclonal anti-HSP27 IgG (Abcam ab79868); 1:5000 rabbit polyclonal anti-αβ-crystallin IgG (ThermoFisher PA1-16,951); and 1:1000 rabbit polyclonal anti-cytochrome c IgG (Abcam ab90529). After being washed 3 times for 10 min with PBST solution, membranes were incubated with the following secondary antibody preparations for one hour at room temperature: goat anti-mouse IgG (H + L) horseradish peroxidase conjugate (dilution of 1:20,000 for desmin, troponin T, and calpain-1; dilution of 1:5000 for HSP27; Bio-Rad) or goat anti-rabbit IgG peroxidase conjugated (dilution of 1:5000 for cytochrome c and αβ-crystallin, ThermoFisher Scientific). After three 10-min washes, ECL Western blotting reagents (ThermoFisher Scientific) were applied to visualize protein bands (UVP GelDoc-It). Intensity volume of each band was measured with UVP VisionworksLS Analysis Software (UVP, LLC; Upland, CA, USA) and were compared with an internal reference to normalize data and quantification.

### Mitochondrial membrane permeability

MMP change is among the key features representing cellular apoptosis^8^. MMP was determined to evaluate the extent of structural integrity loss of mitochondria membranes in LD and PM samples from different production cycles and aging times. The assay isolated mitochondria from LD and PM muscles in both production replicates. Mitochondria isolation was in accordance with Cuillerier et al. (2017) with minor modifications^45^. Muscle samples were minced and transferred to equal volume of pre-chilled isolation buffer (300 mM sucrose, 10 mM Tris–HCl, 1 mM EDTA, pH 7.2). After 20 s homogenizing, the homogenate was centrifuged at 1000×*g* for 10 min at 4 °C for two times, supernatant was each collected. The resulting supernatant was then centrifuged at 8000 × g for 10 min at 4 °C, and the precipitant pellet was kept, and re-suspended in pre-chilled suspension buffer (300 mM sucrose, 10 mM Tris–HCl, 1 mM EDTA, pH 7.2). The suspension was centrifuged at 8000×*g* for 10 min at 4 °C, with the final pellet re-suspended in suspension buffer. The final mitochondria suspension was diluted to a protein concentration of 0.5 mg/mL and incubated for 3 min in water bath at 25 °C. Mitochondrial membrane permeability was determined by measuring absorbance value at 540 nm using the UV spectrophotometer. A higher membrane integrity held more abundant membrane proteins, mainly TCA and ETC enzymes, leading to increased absorption peak. As such, higher numerical measurements indicated more integrated membrane structure, and therefore lower MMP.

### Sample preparation and extraction for metabolomics

LD and PM samples were aged for 1 day (1 g in weight) and were homogenized with 3 mL of methanol for 30 s. The homogenate was shaken for 2 h and centrifuged at 4 °C at 16,000×*g*. Supernatant was collected and was evaporated to dryness with a vacuum centrifuge. Dried muscle extracts were oximated with 50 μL methoxyamine hydrochloride in pyridine at 30 °C for 90 min, and then added to 50 μL of the derivatizing agent, *N*-methyl-*N*-trimethylsilyl-trifluoroacetamide, and incubated at 37 °C for 30 min.

### GC-TOF–MS analysis

The metabolomics analysis was conducted by following the procedure described in Jung et al. (2015) using an Agilent 7890A GC system equipped with an Agilent 7693 autosampler coupled to a Pegasus TOF–MS detector^46^. The separation used an Agilent HP-5MS capillary column, which has an internal diameter of 0.25 mm, a film thickness of 0.25 µm, and a length of 30 m. The carrier gas was chromatographic-grade helium with a constant flow of 1.0 mL/min. The oven temperature was held at 75 °C for 2 min, increased to 300 °C at a rate of 15 °C/min, then maintained at 300 °C for 3 min. A full scan was run at the electron impact mode set to 70 eV ionization energy. The injector line temperature was 250 °C and the transfer line temperature was 240 °C. The GC-TOF–MS used 1 µl of reactant. A pooled quality control sample was analyzed at an interval of eight sample analysis.

The mass-spectrometry data files were converted to CDF format, processed using the metAlign software package to obtain a data matrix containing retention times, accurate masses, and then normalized peak intensities, using sample names and peak area information as variables. For multivariate statistical analysis, the resulting data metric was processed with SIMCA (SIMCAP-P + software version 12.0, Umetrics, Umea, Sweden). The aligned peaks were confirmed in the original chromatograms and were positively or tentatively identified using either commercial standard compounds in comparison with the mass spectra and retention time or on the basis of the NIST mass spectral database, in-house library, and references for GC-TOF–MS.

### Statistical analysis

The experimental design of this study was a complete randomized block design with split plot factors. Production replicates were served as block factor, muscle type effect (LD and PM) as the whole plot, and aging time effect (1 day and 7 days of aging) as a subplot. Animals were considered as a random effect. Apoptotic and proteolytic attributes were analyzed by the PROC MIXED procedure of SAS 9.4 software (SAS Institute Inc.). Least squares means were separated using the PDIFF option of LSMEANS. Significance level were set as α = 0.05 or less. For metabolomics data analysis, principal component analysis (PCA) and partial least squares discriminate analysis (PLS-DA) modeling were performed by SIMCA and PASW Statistics 18. In PLS-DA, the discriminated variables were selected based on variable importance in the projection value and checked with P-value from one-way ANOVA. The metabolic pathway was modified from the KEGG database (http://www.genome.jp/kegg/)^47^.

## Supplementary Information

Supplementary Figures.

## References

[1] Kim YHB (2018). Understanding postmortem biochemical processes and post-harvest aging factors to develop novel smart-aging strategies. Meat Sci..

[2] Kemp CM, Sensky PL, Bardsley RG, Buttery PJ, Parr T (2010). Tenderness—An enzymatic view. Meat Sci..

[3] Carlson K (2017). Postmortem protein degradation is a key contributor to fresh pork loin tenderness. J. Anim. Sci..

[4] Kemp, C. M. & Parr, T. Advances in apoptotic mediated proteolysis in meat tenderisation. Meat Sci. **92**, 252–259, 10.1016/j.meatsci.2012.03.013 (2012). 10.1016/j.meatsci.2012.03.013 22546815

[5] Ouali A (2013). Biomarkers of meat tenderness: Present knowledge and perspectives in regards to our current understanding of the mechanisms involved. Meat Sci..

[6] Ouali A (2006). Revisiting the conversion of muscle into meat and the underlying mechanisms. Meat Sci..

[7] Wang L-L (2018). Study on the effect of reactive oxygen species-mediated oxidative stress on the activation of mitochondrial apoptosis and the tenderness of yak meat. Food Chem..

[8] Wang L-L, Han L, Ma X-L, Yu Q-L, Zhao S-N (2017). Effect of mitochondrial apoptotic activation through the mitochondrial membrane permeability transition pore on yak meat tenderness during postmortem aging. Food Chem..

[9] Lee S, Joo S, Ryu Y (2010). Skeletal muscle fiber type and myofibrillar proteins in relation to meat quality. Meat Sci..

[10] Ma D, Kim YHB (2020). Proteolytic changes of myofibrillar and small heat shock proteins in different bovine muscles during aging: Their relevance to tenderness and water-holding capacity. Meat Sci..

[11] Ma D, Kim Y, Johnson J, Duttlinger A, Guedes J (2019). Effect of L-glutamine supplementation in replacement of antibiotics on meat quality attributes of pigs exposed to transport and weaning stress during different seasons. Meat Muscle Biol..

[12] Ma D (2017). Metabolomics profiling to determine the effect of postmortem aging on color and lipid oxidative stabilities of different bovine muscles. J. Agric. Food Chem..

[13] Subbaraj AK, Kim YHB, Fraser K, Farouk MM (2016). A hydrophilic interaction liquid chromatography–mass spectrometry (HILIC–MS) based metabolomics study on colour stability of ovine meat. Meat Sci..

[14] Smith MA, Schnellmann RG (2012). Calpains, mitochondria, and apoptosis. Cardiovasc. Res..

[15] Altznauer F, Conus S, Cavalli A, Folkers G, Simon H-U (2004). Calpain-1 regulates Bax and subsequent Smac-dependent caspase-3 activation in neutrophil apoptosis. J. Biol. Chem..

[16] Wang KK (1998). Caspase-mediated fragmentation of calpain inhibitor protein calpastatin during apoptosis. Arch. Biochem. Biophys..

[17] Zheng, D., Wang, G., Li, S., Fan, G.-C. & Peng, T. Calpain-1 induces endoplasmic reticulum stress in promoting cardiomyocyte apoptosis following hypoxia/reoxygenation. *Biochim. Biophys. Acta (BBA)-Mol. Basis Dis*. **1852**, 882–892, 10.1016/j.bbadis.2015.01.019 (2015). 10.1016/j.bbadis.2015.01.019 PMC4418534 25660447

[18] Ke Y (2017). Effects of muscle-specific oxidative stress on cytochrome c release and oxidation-reduction potential properties. J. Agric. Food Chem..

[19] Rowe LJ, Maddock KR, Lonergan SM, Huff-Lonergan E (2004). Oxidative environments decrease tenderization of beef steaks through inactivation of μ-calpain. J. Anim. Sci..

[20] Stangel M (1996). H2O2 and nitric oxide-mediated oxidative stress induce apoptosis in rat skeletal muscle myoblasts. J. Neuropathol. Exp. Neurol..

[21] Koves TR (2008). Mitochondrial overload and incomplete fatty acid oxidation contribute to skeletal muscle insulin resistance. Cell Metab..

[22] Unger, R. H. & Orci, L. Lipoapoptosis: its mechanism and its diseases. *Biochim. Biophys. Acta (BBA)-Mol. Cell Biol. Lipids* **1585**, 202–212, 10.1016/s1388-1981(02)00342-6 (2002). 10.1016/s1388-1981(02)00342-6 12531555

[23] Kusminski CM, Shetty S, Orci L, Unger RH, Scherer PE (2009). Diabetes and apoptosis: lipotoxicity. Apoptosis.

[24] Morino K, Petersen KF, Shulman GI (2006). Molecular mechanisms of insulin resistance in humans and their potential links with mitochondrial dysfunction. Diabetes.

[25] Turpin SM, Lancaster GI, Darby I, Febbraio MA, Watt MJ (2006). Apoptosis in skeletal muscle myotubes is induced by ceramides and is positively related to insulin resistance. Am. J. Physiol. Endocrinol. Metab..

[26] Goldspink D (1977). The influence of immobilization and stretch on protein turnover of rat skeletal muscle. J. Physiol..

[27] Katunuma N, Okada M, Nishii Y (1966). Regulation of the urea cycle and TCA cycle by ammonia. Adv. Enzyme Regul..

[28] Fernie AR, Carrari F, Sweetlove LJ (2004). Respiratory metabolism: Glycolysis, the TCA cycle and mitochondrial electron transport. Curr. Opin. Plant Biol..

[29] James AM, Collins Y, Logan A, Murphy MP (2012). Mitochondrial oxidative stress and the metabolic syndrome. Trends Endocrinol. Metab..

[30] Yoshizawa F (2004). Regulation of protein synthesis by branched-chain amino acids in vivo. Biochem. Biophys. Res. Commun..

[31] Bridi R (2003). Induction of oxidative stress in rat brain by the metabolites accumulating in maple syrup urine disease. Int. J. Dev. Neurosci..

[32] Holeček M (2018). Branched-chain amino acids in health and disease: Metabolism, alterations in blood plasma, and as supplements. Nutr. Metab..

[33] Fernandes CG (2010). Experimental evidence that phenylalanine provokes oxidative stress in hippocampus and cerebral cortex of developing rats. Cell. Mol. Neurobiol..

[34] Martínez Y (2017). The role of methionine on metabolism, oxidative stress, and diseases. Amino Acids.

[35] Wade AM, Tucker HN (1998). Antioxidant characteristics of L-histidine. J. Nutr. Biochem..

[36] Chen L (2018). Glycine transporter-1 and glycine receptor mediate the antioxidant effect of glycine in diabetic rat islets and INS-1 cells. Free Radical Biol. Med..

[37] Min Y, Liu S, Qu Z, Meng G, Gao Y (2017). Effects of dietary threonine levels on growth performance, serum biochemical indexes, antioxidant capacities, and gut morphology in broiler chickens. Poult. Sci..

[38] Duan J (2016). Dietary supplementation with L-glutamate and L-aspartate alleviates oxidative stress in weaned piglets challenged with hydrogen peroxide. Amino Acids.

[39] Pi D (2014). Dietary supplementation of aspartate enhances intestinal integrity and energy status in weanling piglets after lipopolysaccharide challenge. J. Nutr. Biochem..

[40] Grosser N (2004). Antioxidant action of L-alanine: Heme oxygenase-1 and ferritin as possible mediators. Biochem. Biophys. Res. Commun..

[41] Vaiva G (2004). Low posttrauma GABA plasma levels as a predictive factor in the development of acute posttraumatic stress disorder. Biol. Psychiat..

[42] Duttlinger, A. W., Kpodo, K. R., Lay Jr, D. C., Richert, B. T. & Johnson, J. S. Replacing dietary antibiotics with 0.20% L-glutamine in swine nursery diets: Impact on health and productivity of pigs following weaning and transport. *J. Anim. Sci.* **97**, 2035–2052, 10.1093/jas/skz098 (2019). 10.1093/jas/skz098 PMC6488329 30924491

[43] Grum CM, Simon RH, Dantzker DR, Fox IH (1985). Evidence for adenosine triphosphate degradation in critically-ill patients. Chest.

[44] Buonocore G (2002). Oxidative stress in preterm neonates at birth and on the seventh day of life. Pediatr. Res..

[45] Cuillerier A (2017). Loss of hepatic LRPPRC alters mitochondrial bioenergetics, regulation of permeability transition and trans-membrane ROS diffusion. Hum. Mol. Genet..

[46] Jung, E. S. *et al*. A metabolomics approach shows that catechin-enriched green tea attenuates ultraviolet B-induced skin metabolite alterations in mice. *Metabolomics* **11**, 861–871, 10.1007/s11306-014-0743-x (2015).

[47] Kanehisa M, Goto S (2000). KEGG: Kyoto encyclopedia of genes and genomes. Nucleic Acids Res..

