# Supplementary material for: Elucidating the involvement of apoptosis in postmortem proteolysis in porcine muscles from two production cycles using metabolomics approach
Source: Sci Rep. 2021 Feb 10;11:3465. doi: 10.1038/s41598-021-82929-3 (PMC7876139; doi:10.1038/s41598-021-82929-3)
Supplement: Supplementary file 1 — Supplementary Figures. [file 41598_2021_82929_MOESM1_ESM.pdf]

Supplementary materials (whole western-blot images)

for

Elucidating the involvement of apoptosis in postmortem proteolysis in porcine muscles from two production cycles using metabolomics approach

Danyi Ma[1, †], Dong Ho Suh[2, †], Jiaying Zhang[1], Yufan Chao[1], Alan W. Duttlinger[1,3], Jay S. Johnson[3], Choong Hwan Lee[4,*], Yuan H. Brad Kim[1,*]

[1] Department of Animal Sciences, Purdue University, West Lafayette, IN 47907, USA.
[2] Department of Bioscience and Biotechnology, Konkuk University, Seoul, 05029, South Korea.
[3] USDA-ARS Livestock Behavior Research Unit, West Lafayette, IN 47907, USA.
[4]Research Institute for Bioactive-Metabolome Network, Konkuk University, Seoul, 05029, South Korea
* bradkim@purdue.edu, chlee123@konkuk.ac.kr
[†]these authors contributed equally to this work

Order

Page 2-8 Desmin

Page 9-13 Troponin-T

Page 14-19 Calpain-1

Page 20-21 HSP27

Page 22-24 αβ-crystallin

Page 25-26 cytochrome c

All images were non-cropped by any post-processing software. Membranes were pre-cut prior to primary antibody hybridisation.

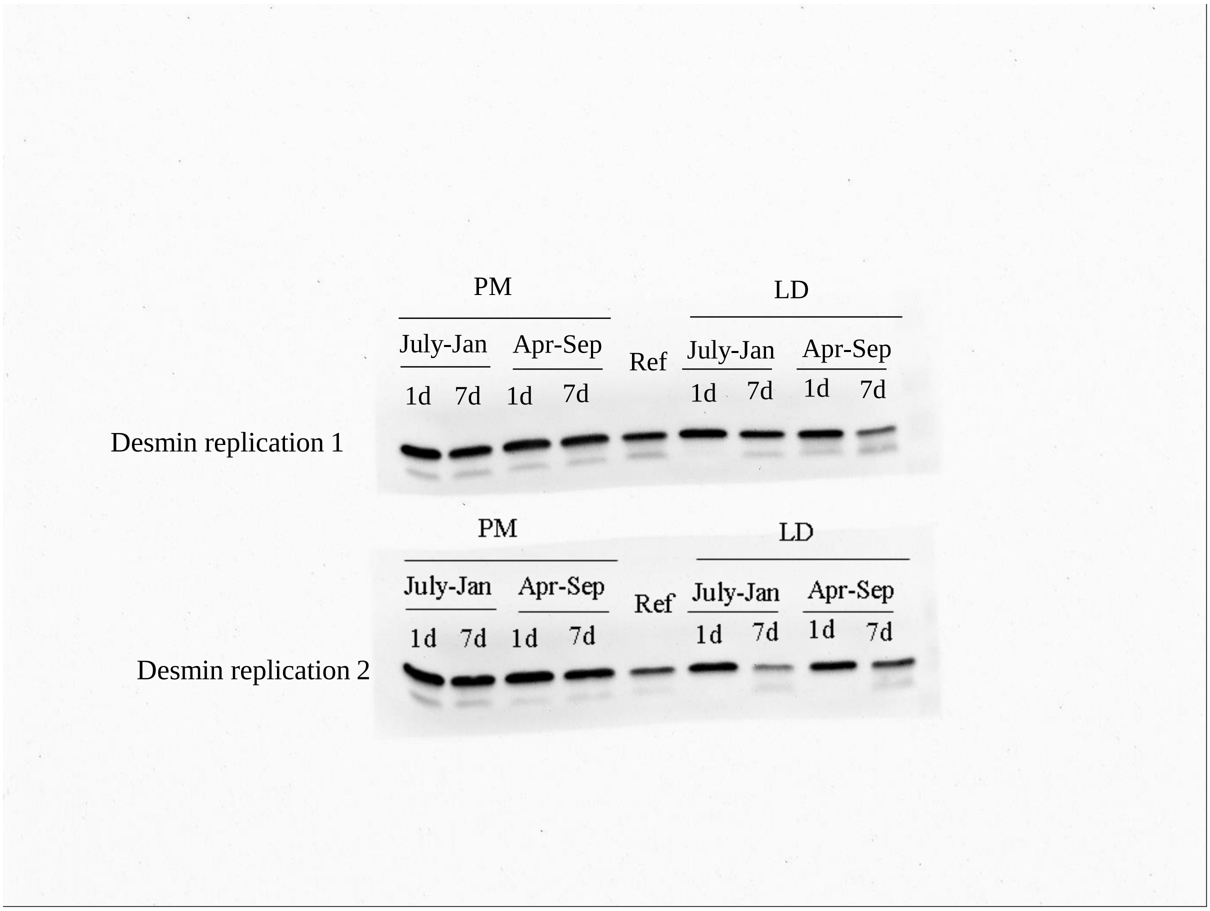
PM
LD
July-Jan
Apr-Sep
Ref
July-Jan
Apr-Sep
1d 7d 1d 7d
1d 7d 1d 7d
Desmin replication 1
PM
LD
July-Jan
Apr-Sep
Ref
July-Jan
Apr-Sep
1d 7d 1d 7d
1d 7d 1d 7d
Desmin replication 2

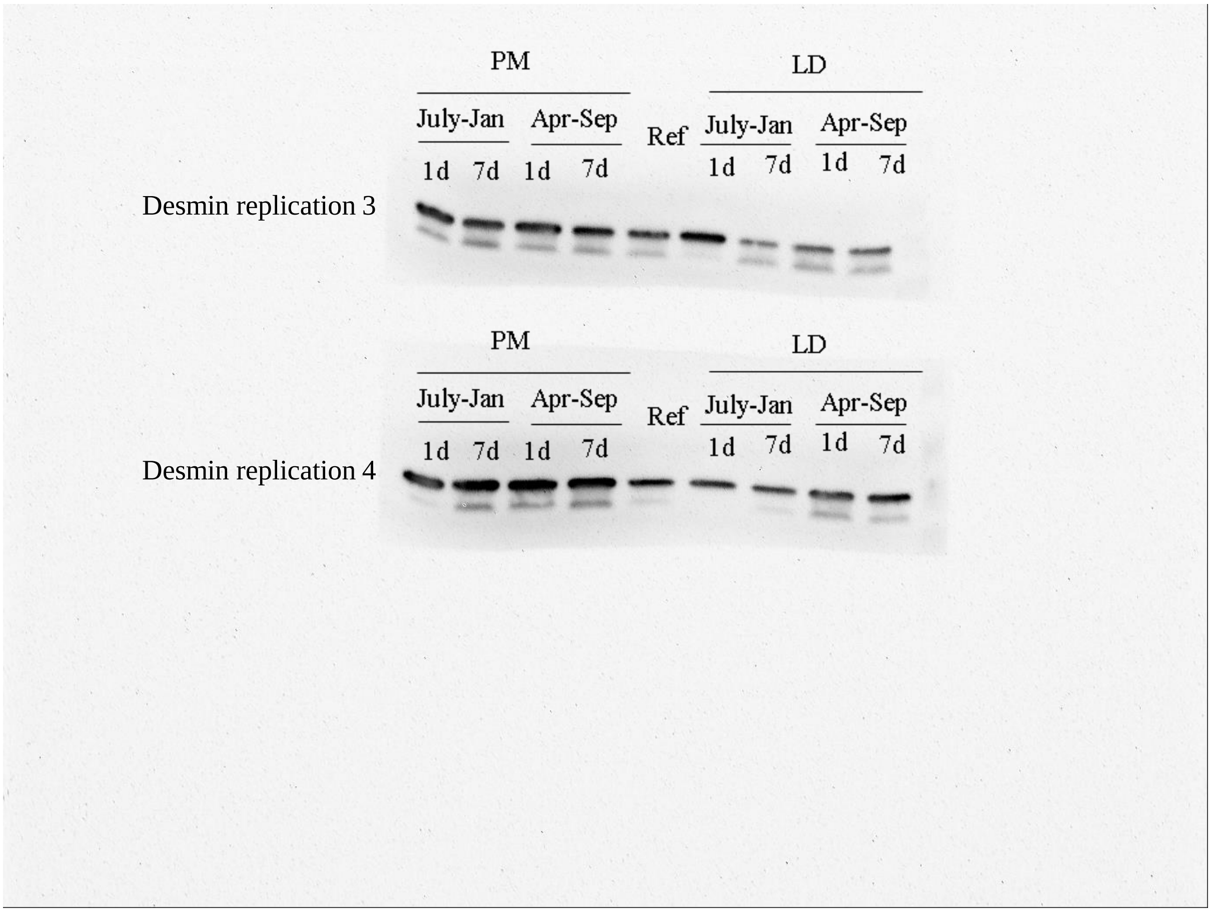
PM
LD
July-Jan
Apr-Sep
Ref
July-Jan
Apr-Sep
1d 7d 1d 7d
1d 7d 1d 7d
Desmin replication 3
PM
LD
July-Jan
Apr-Sep
Ref
July-Jan
Apr-Sep
1d 7d 1d 7d
1d 7d 1d 7d
Desmin replication 4

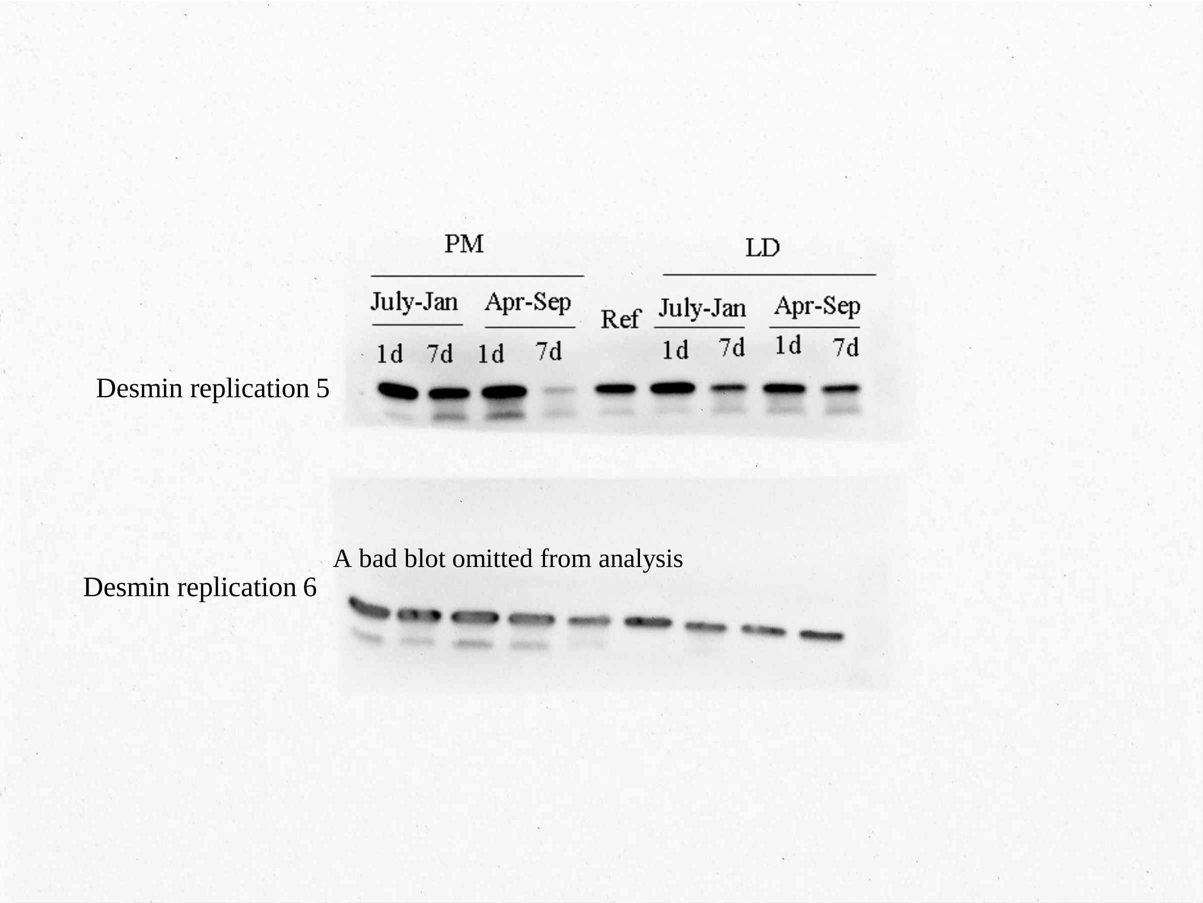
PM
LD
July-Jan
Apr-Sep
Ref
July-Jan
Apr-Sep
1d 7d 1d 7d
1d 7d 1d 7d
Desmin replication 5
A bad blot omitted from analysis
Desmin replication 6

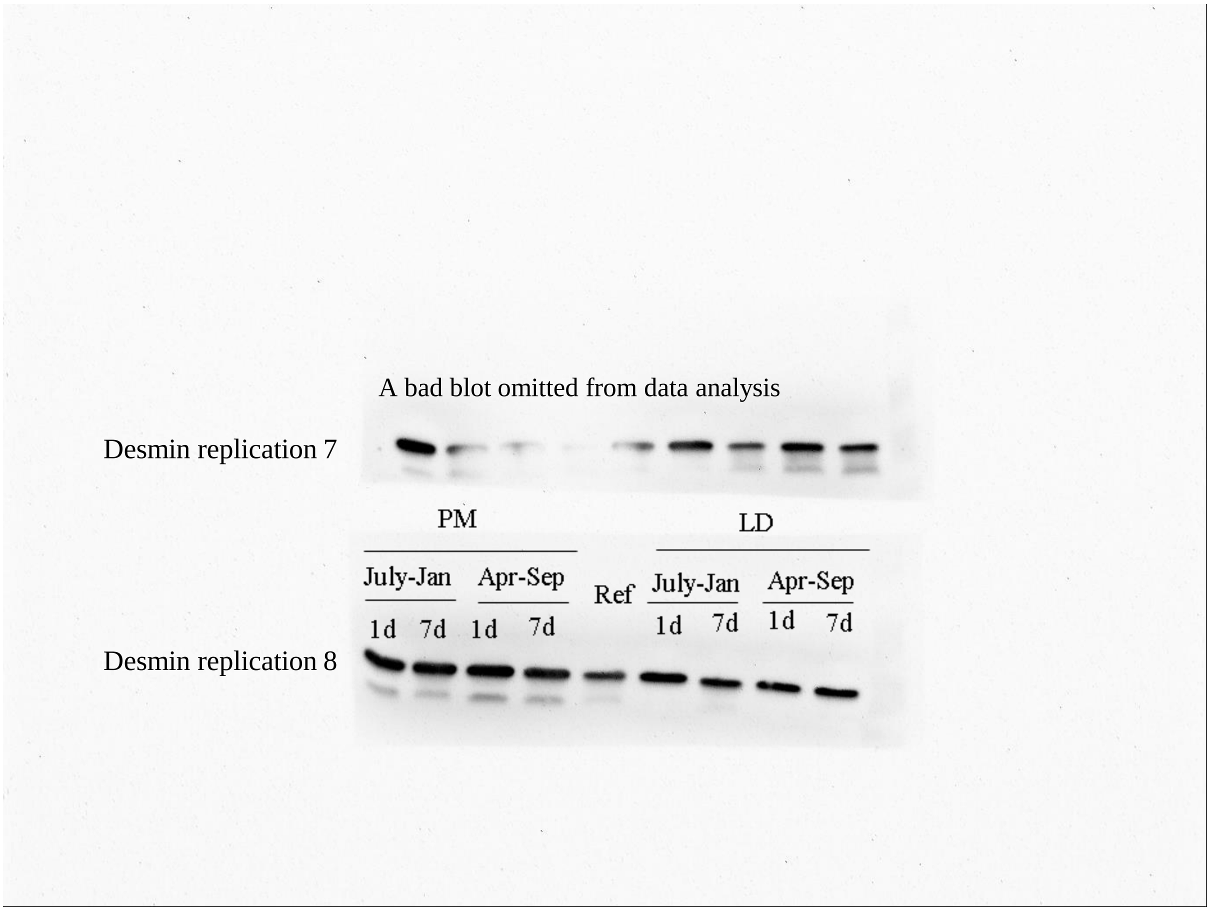
A bad blot omitted from data analysis
Desmin replication 7
PM
LD
July-Jan
Apr-Sep
Ref
July-Jan
Apr-Sep
1d 7d 1d 7d
1d 7d 1d 7d
Desmin replication 8

Desmin replication 9

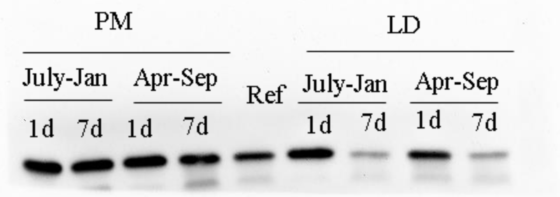

Desmin replication 10

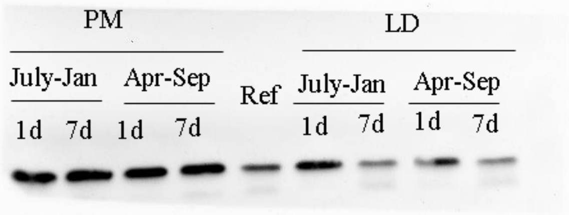

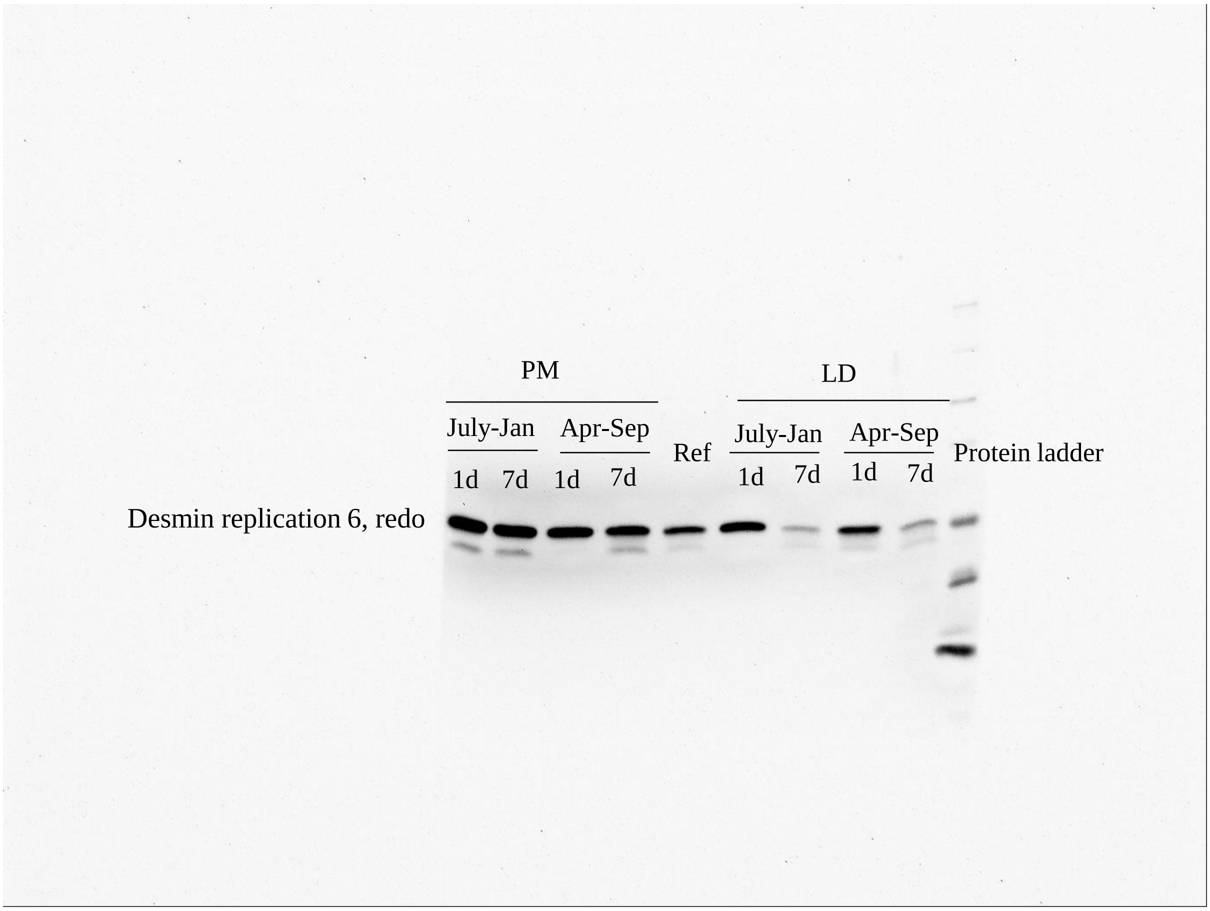
PM
LD
July-Jan
Apr-Sep
Ref
July-Jan
Apr-Sep
Protein ladder
1d
7d
1d
7d
1d
7d
1d
7d
Desmin replication 6, redo

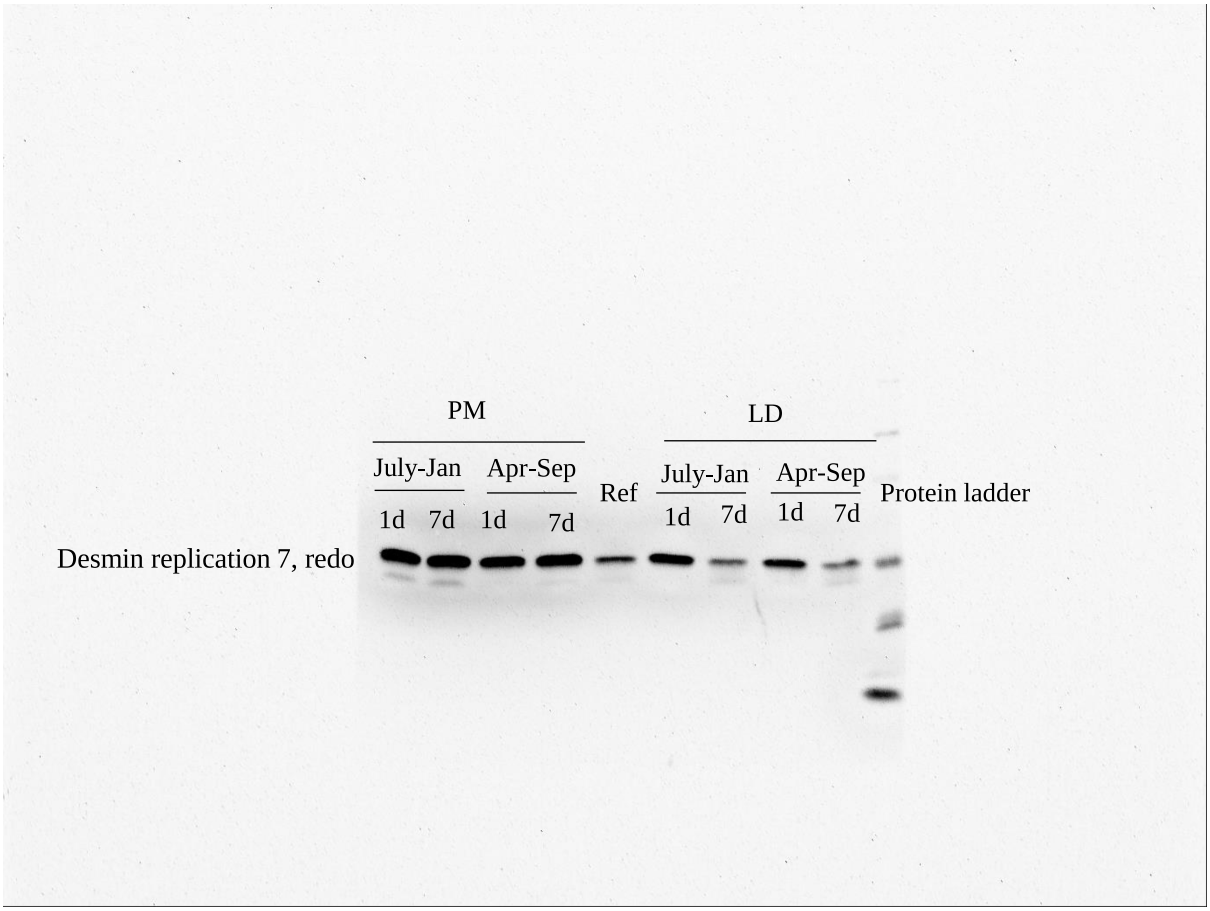
PM
LD
July-Jan
Apr-Sep
Ref
July-Jan
Apr-Sep
Protein ladder
1d
7d
1d
7d
1d
7d
1d
7d
Desmin replication 7, redo

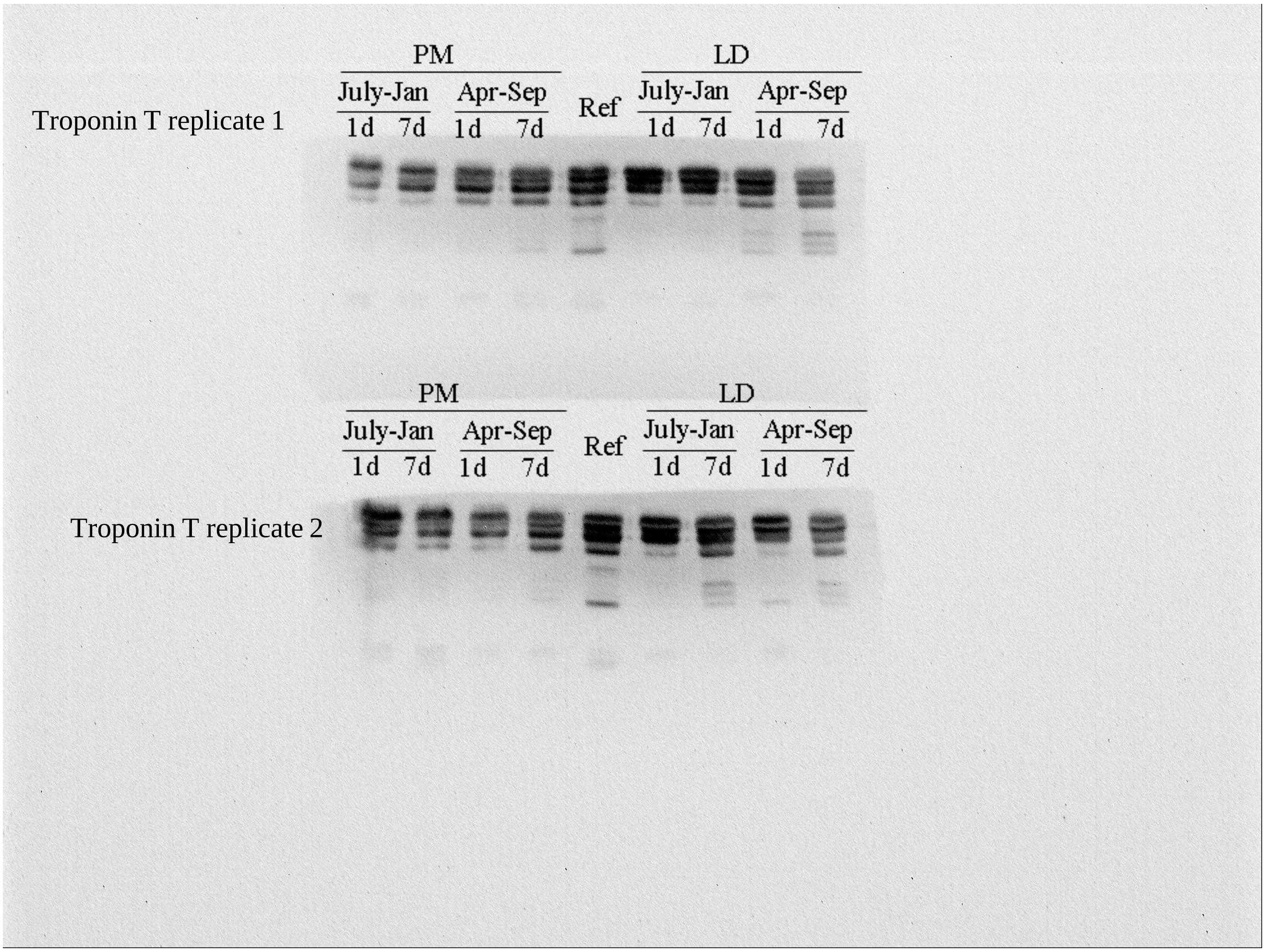
Troponin T replicate 1
PM
LD
July-Jan
Apr-Sep
Ref
July-Jan
Apr-Sep
1d 7d 1d 7d
1d 7d 1d 7d
Troponin T replicate 2
PM
LD
July-Jan
Apr-Sep
Ref
July-Jan
Apr-Sep
1d 7d 1d 7d
1d 7d 1d 7d

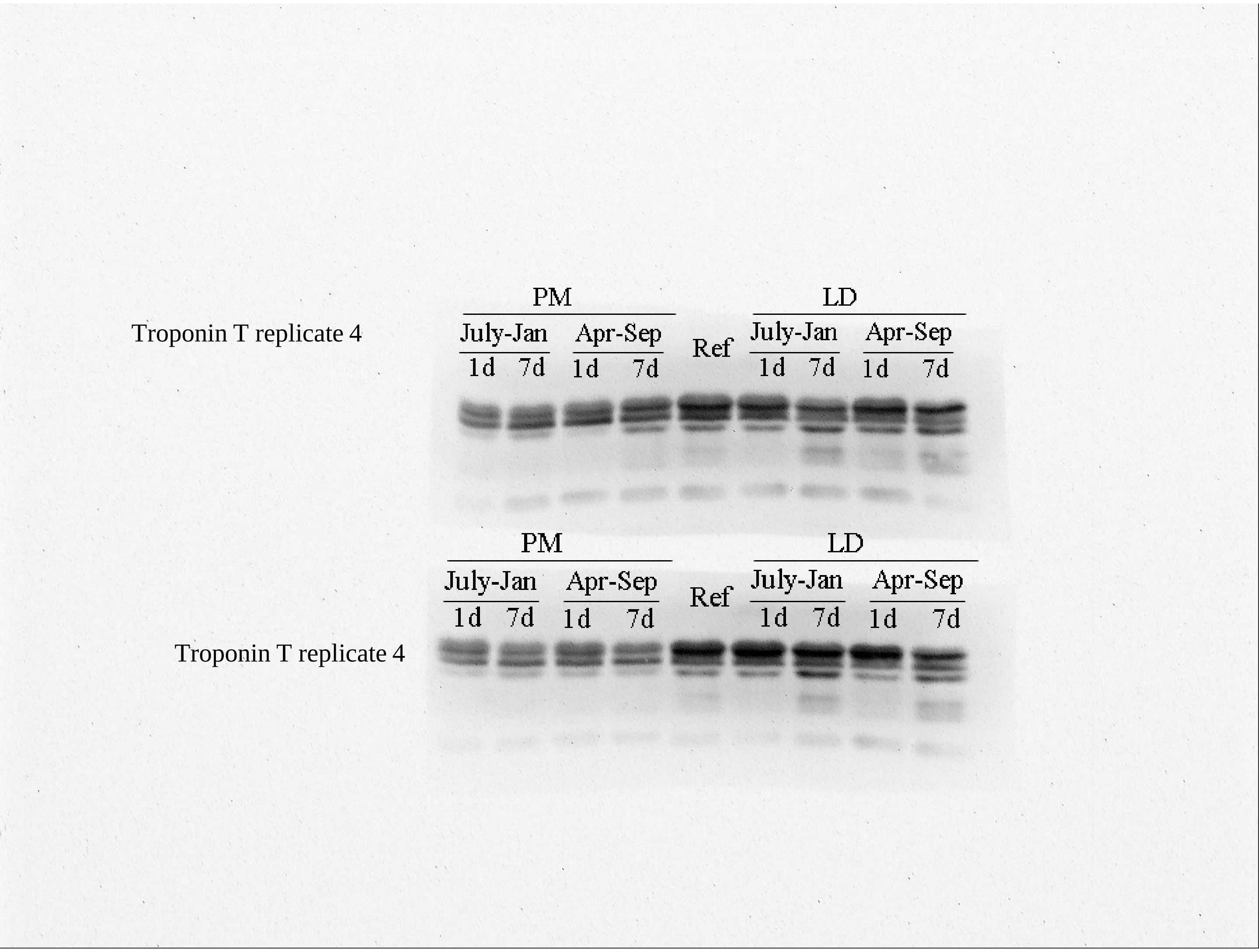
Troponin T replicate 4
PM
LD
July-Jan
Apr-Sep
Ref
July-Jan
Apr-Sep
1d 7d 1d 7d
1d 7d 1d 7d
PM
LD
July-Jan
Apr-Sep
Ref
July-Jan
Apr-Sep
1d 7d 1d 7d
1d 7d 1d 7d
Troponin T replicate 4

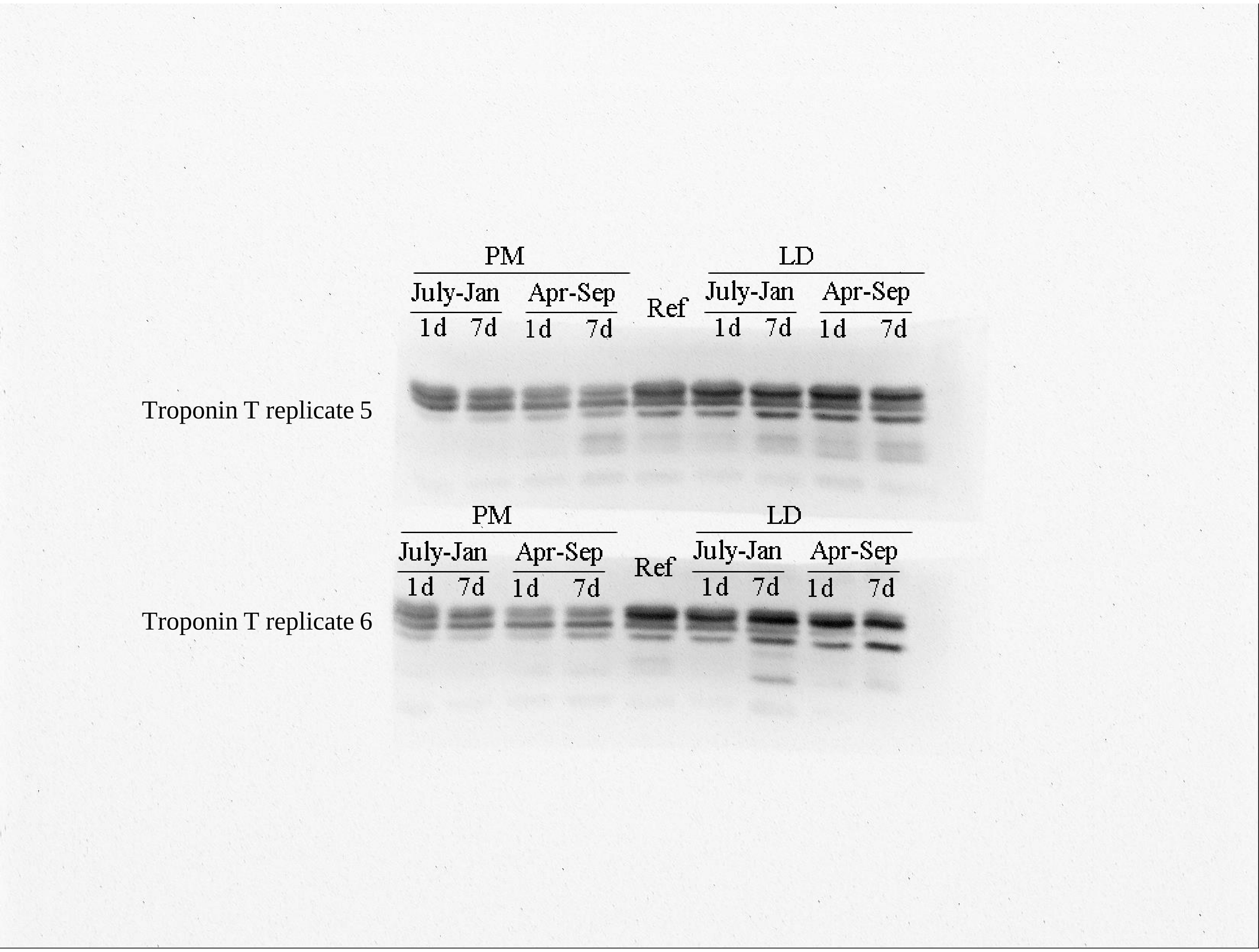
PM
LD
July-Jan
Apr-Sep
Ref
1d
7d
Troponin T replicate 5
Troponin T replicate 6

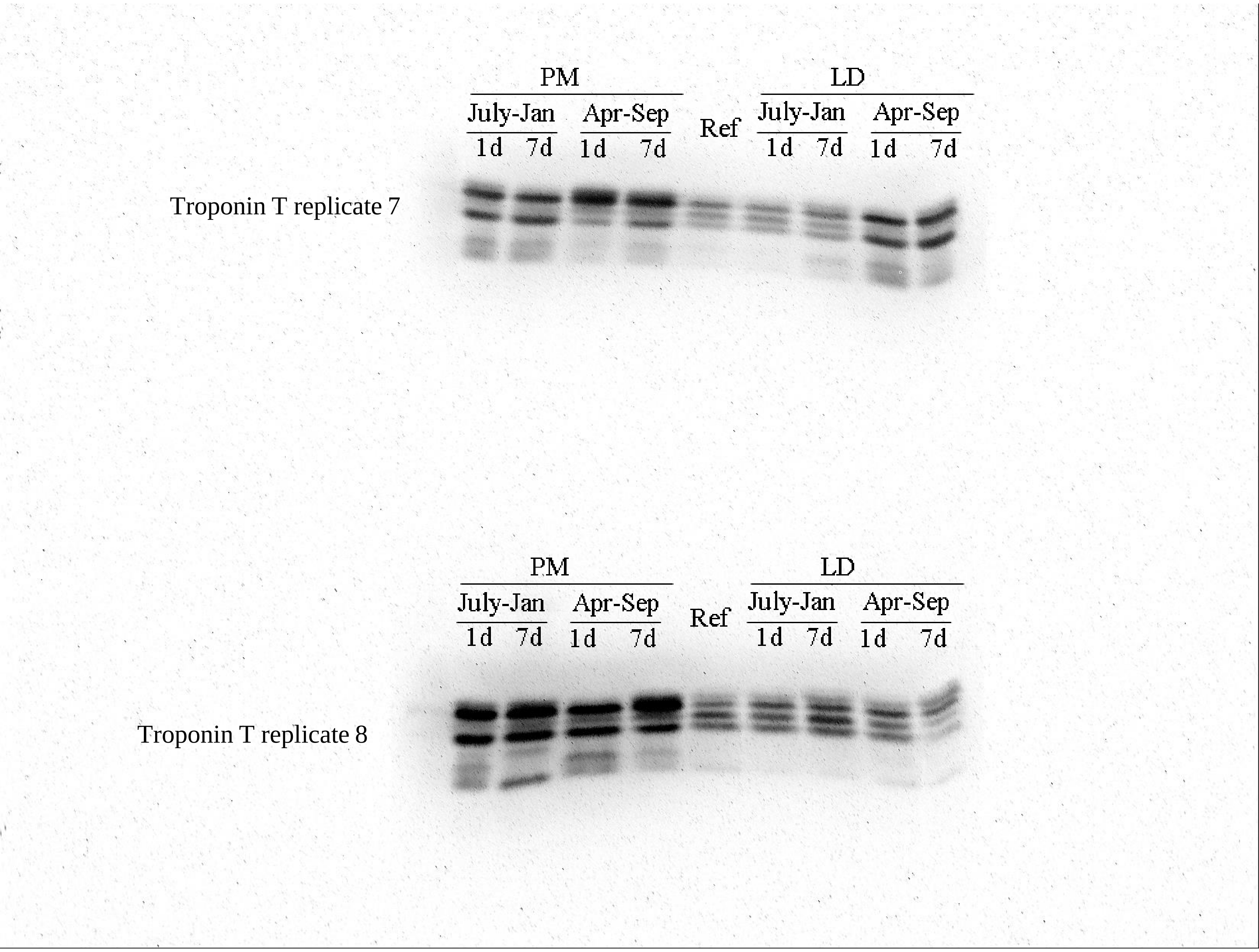
PM
LD
July-Jan
Apr-Sep
Ref
July-Jan
Apr-Sep
1d
7d
1d
7d
1d
7d
1d
7d
Troponin T replicate 7
PM
LD
July-Jan
Apr-Sep
Ref
July-Jan
Apr-Sep
1d
7d
1d
7d
1d
7d
1d
7d
Troponin T replicate 8

Troponin T replicate 9

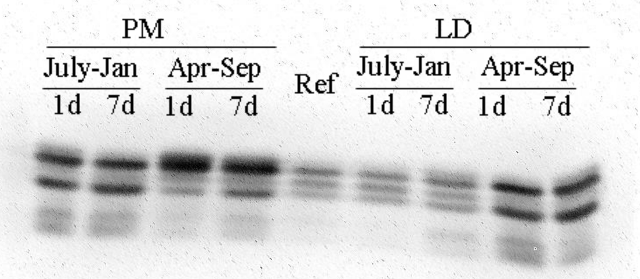

Troponin T replicate 10

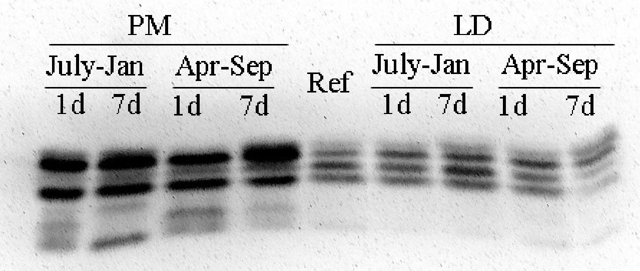

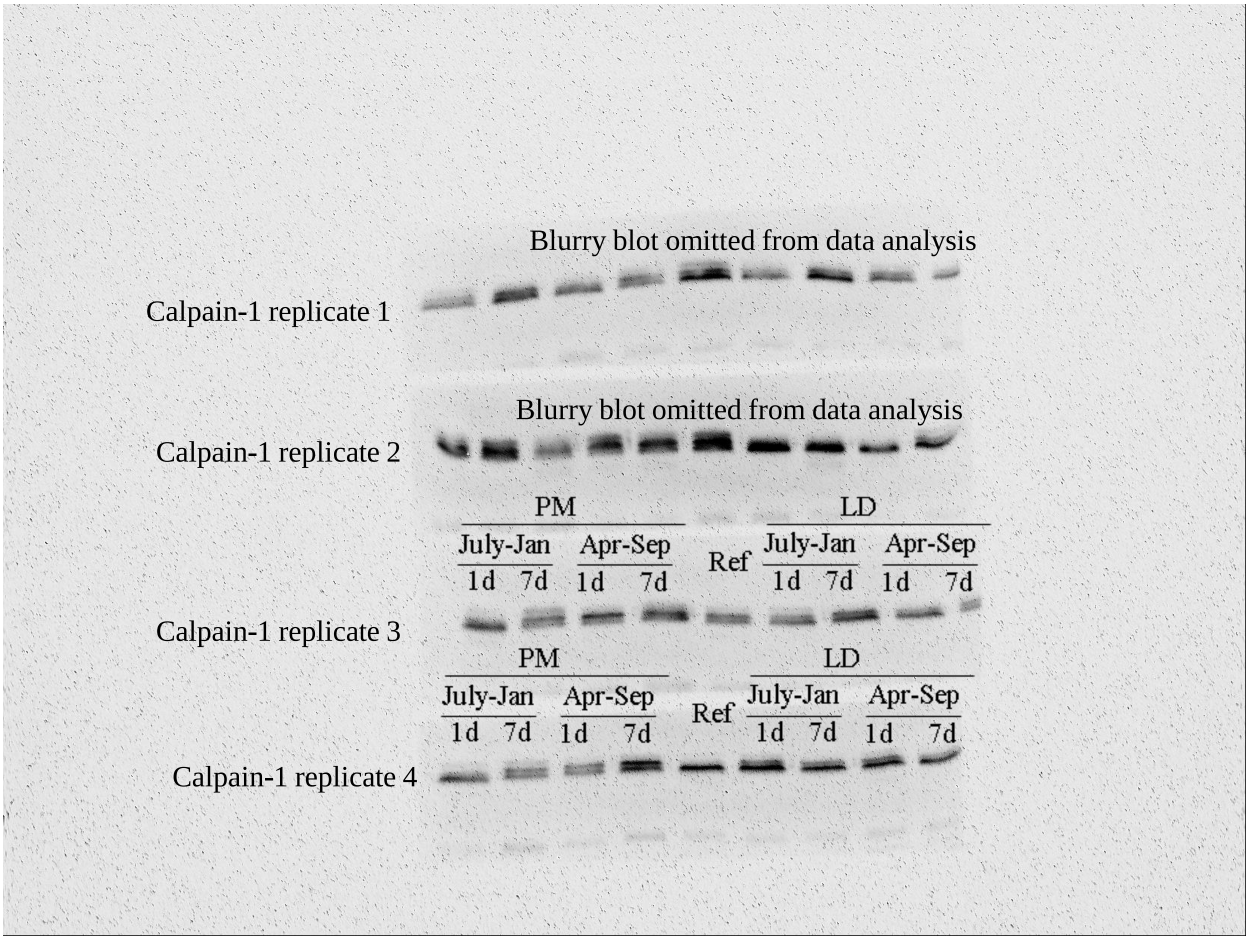

Blurry blot omitted from data analysis
Calpain-1 replicate 1
Blurry blot omitted from data analysis
Calpain-1 replicate 2
PM
LD
July-Jan
Apr-Sep
Ref
July-Jan
Apr-Sep
1d 7d 1d 7d
1d 7d 1d 7d
Calpain-1 replicate 3
PM
LD
July-Jan
Apr-Sep
Ref
July-Jan
Apr-Sep
1d 7d 1d 7d
1d 7d 1d 7d
Calpain-1 replicate 4

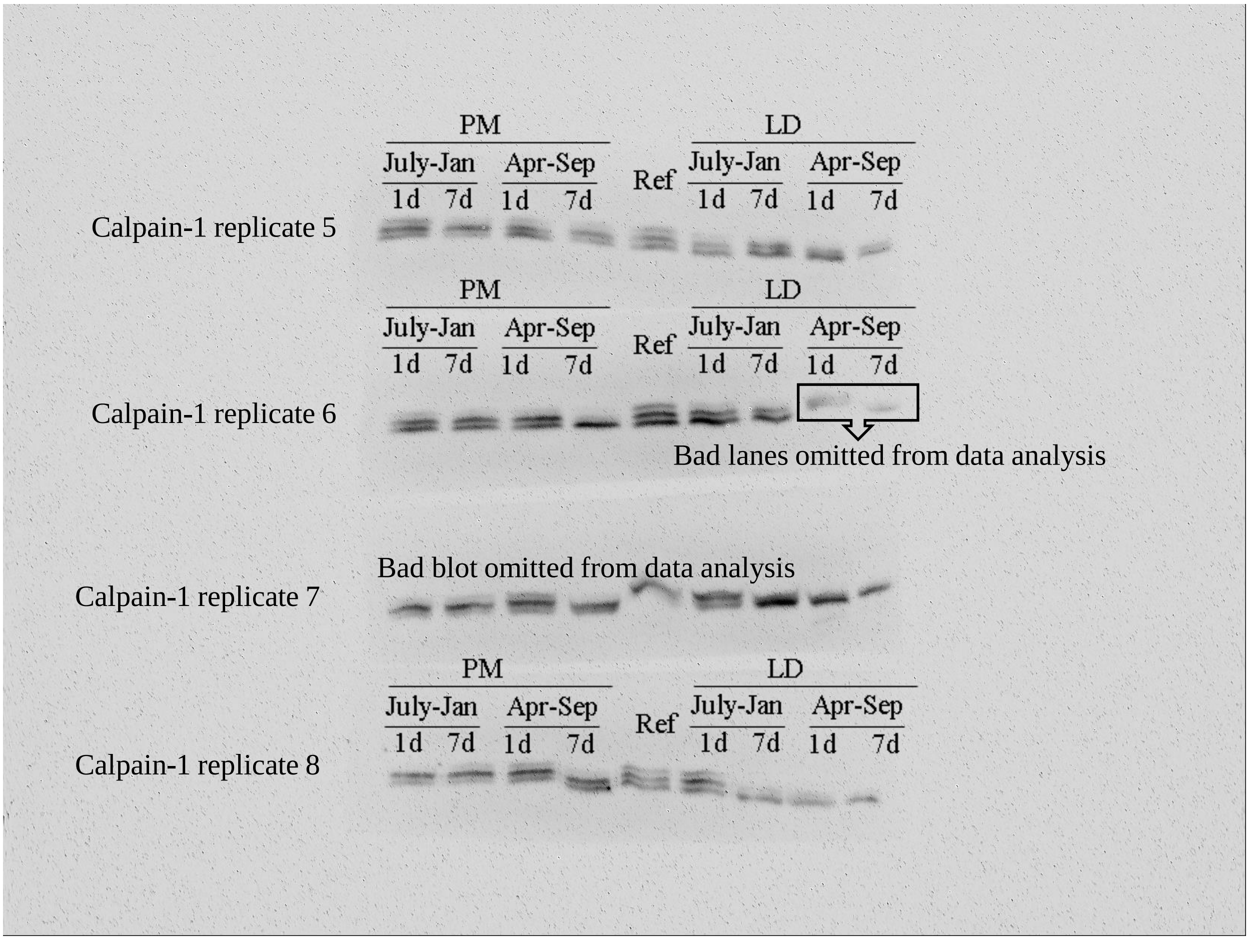
PM
LD
July-Jan
Apr-Sep
Ref
1d 7d 1d 7d
Calpain-1 replicate 5
Calpain-1 replicate 6
Bad lanes omitted from data analysis
Bad blot omitted from data analysis
Calpain-1 replicate 7
Calpain-1 replicate 8

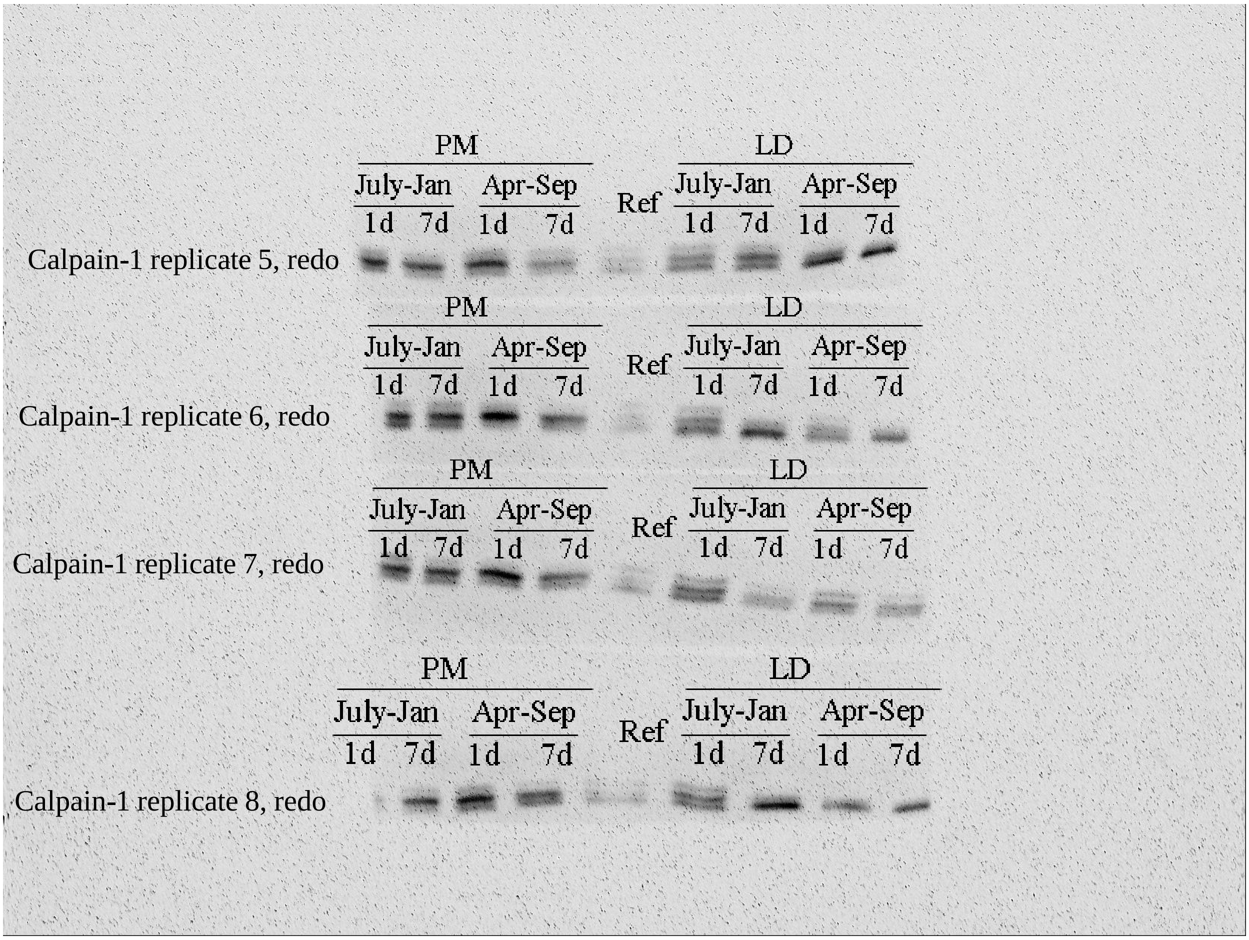
PM
LD
July-Jan
Apr-Sep
Ref
1d
7d
Calpain-1 replicate 5, redo
Calpain-1 replicate 6, redo
Calpain-1 replicate 7, redo
Calpain-1 replicate 8, redo

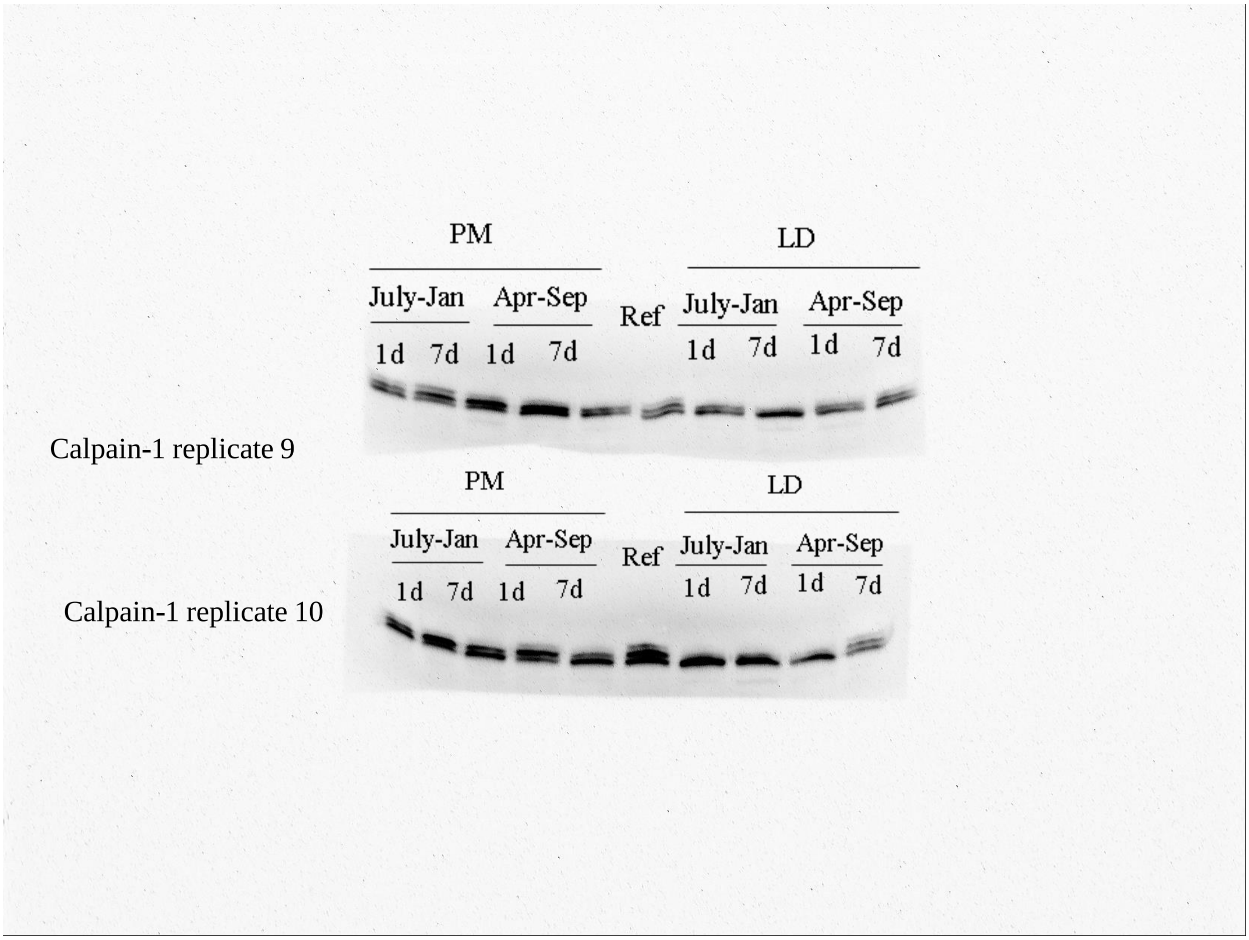
PM
LD
July-Jan
Apr-Sep
Ref
July-Jan
Apr-Sep
1d 7d 1d 7d
1d 7d 1d 7d
Calpain-1 replicate 9
PM
LD
July-Jan
Apr-Sep
Ref
July-Jan
Apr-Sep
1d 7d 1d 7d
1d 7d 1d 7d
Calpain-1 replicate 10

Calpain-1 replicate 1, redo

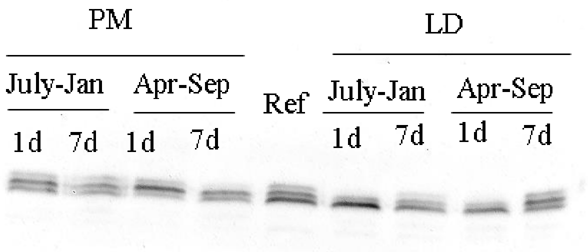

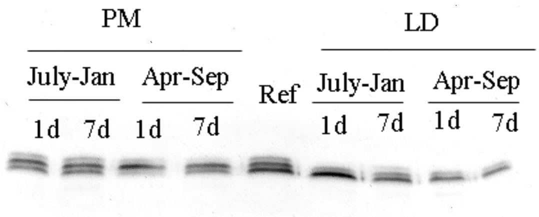

Calpain-1 replicate 2, redo

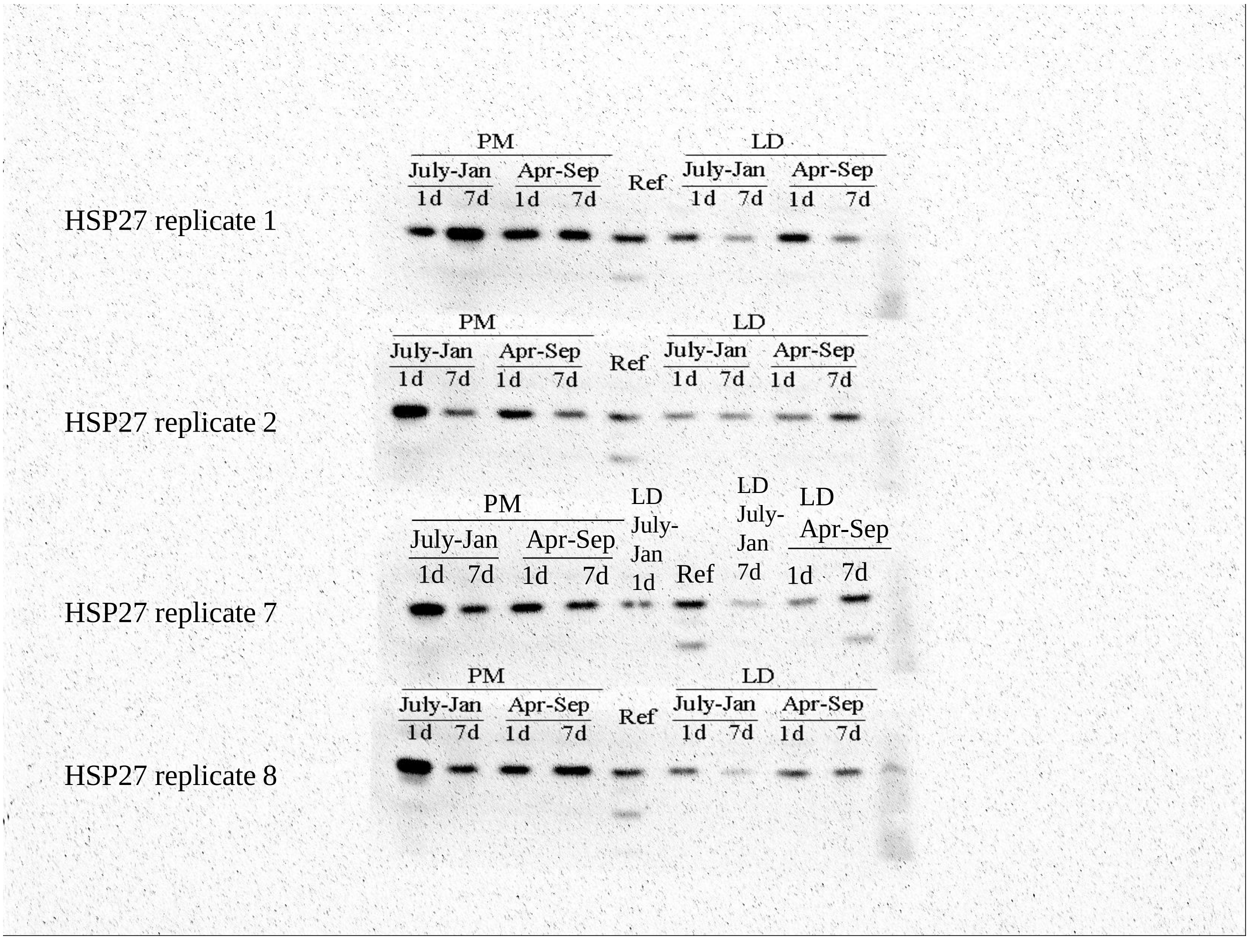

HSP27 replicate 1
PM
LD
July-Jan
Apr-Sep
Ref
1d
7d
HSP27 replicate 2
HSP27 replicate 7
LD July-Jan 1d
LD July-Jan 7d
LD Apr-Sep
HSP27 replicate 8

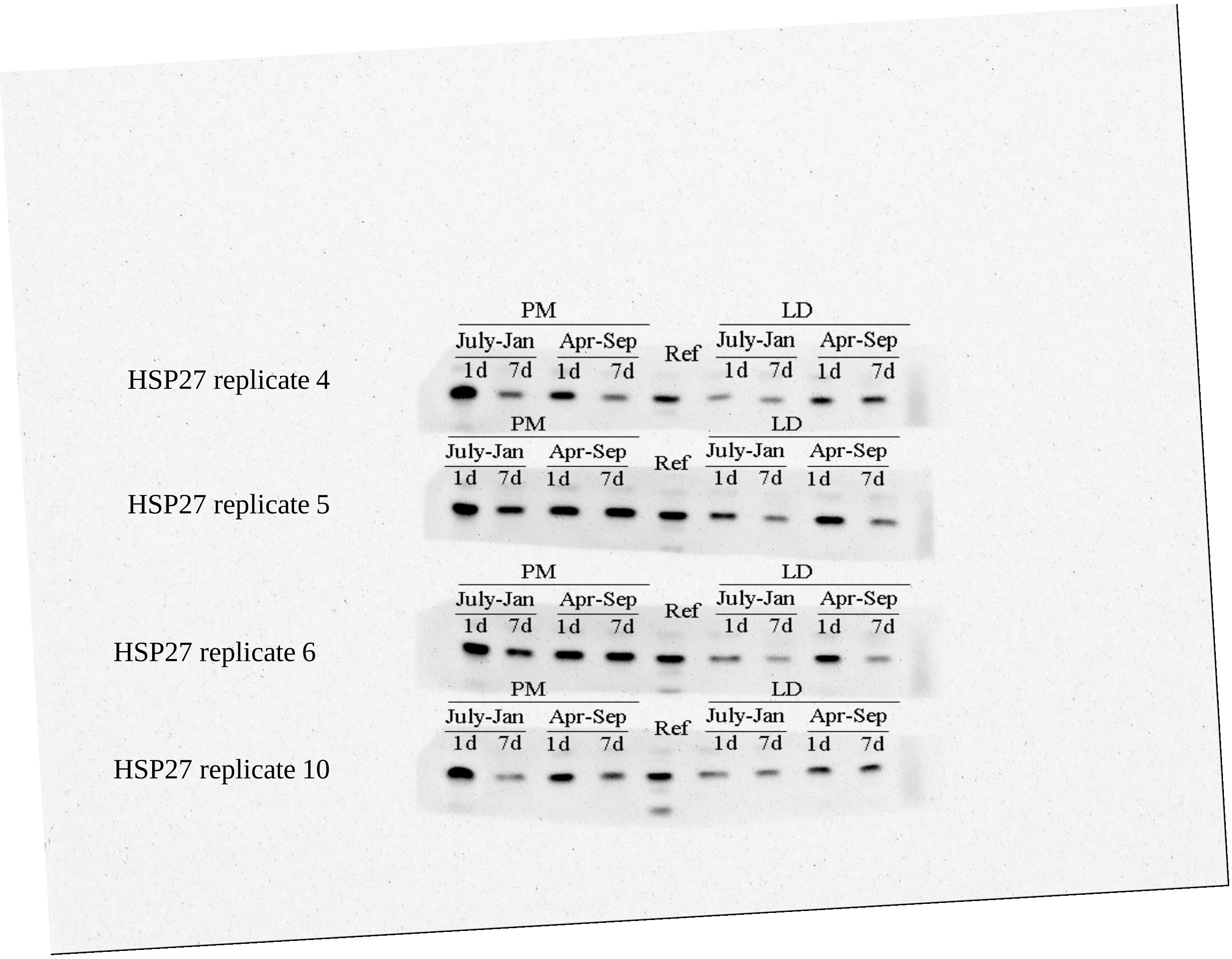
HSP27 replicate 4
HSP27 replicate 5
HSP27 replicate 6
HSP27 replicate 10
PM
LD
July-Jan
Apr-Sep
Ref
1d
7d

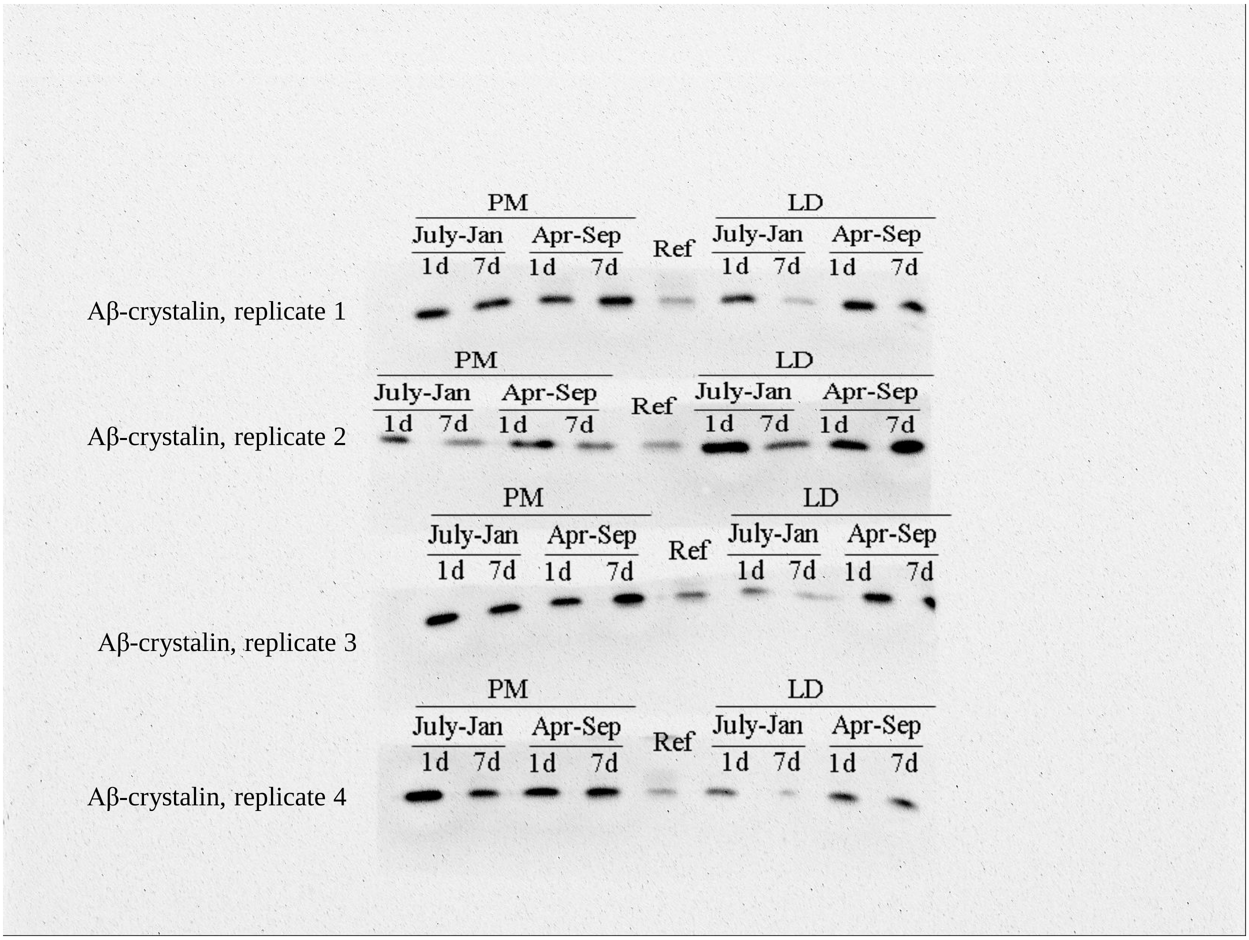
PM
LD
July-Jan
Apr-Sep
Ref
1d
7d
Aβ-crystalin, replicate 1
Aβ-crystalin, replicate 2
Aβ-crystalin, replicate 3
Aβ-crystalin, replicate 4

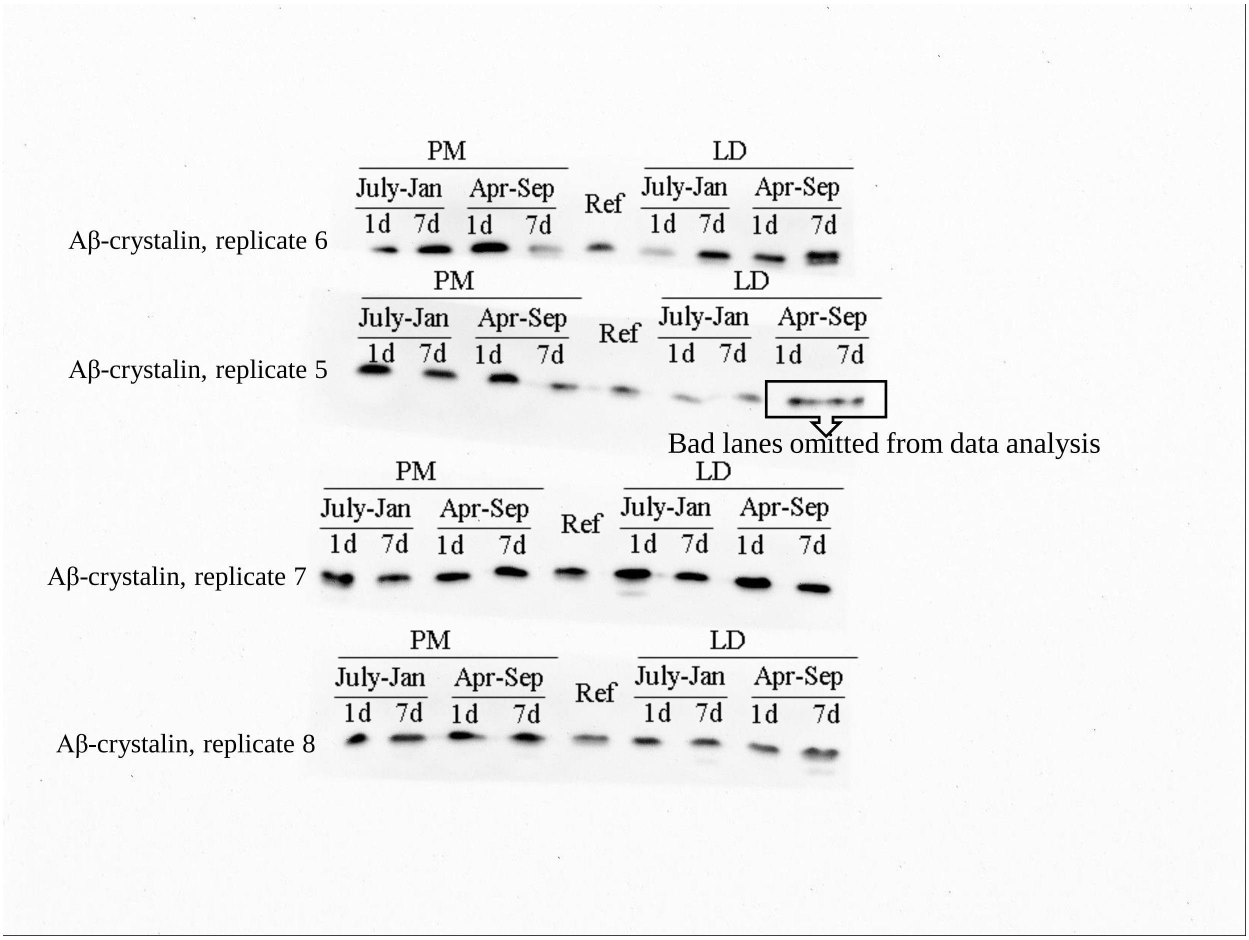
PM
LD
July-Jan
Apr-Sep
Ref
1d
7d
Aβ-crystalin, replicate 6
Aβ-crystalin, replicate 5
Bad lanes omitted from data analysis
Aβ-crystalin, replicate 7
Aβ-crystalin, replicate 8

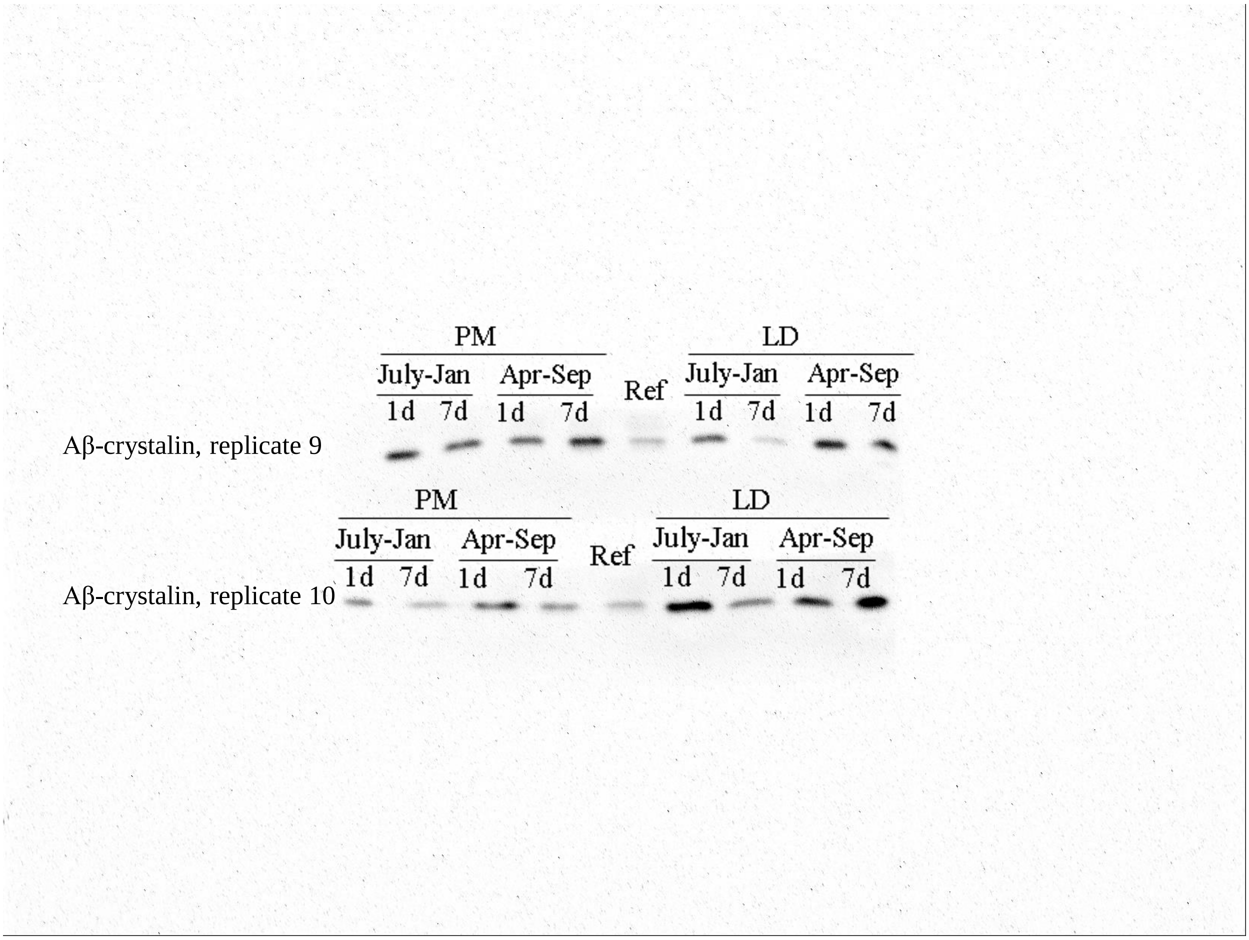
PM
LD
July-Jan
Apr-Sep
Ref
July-Jan
Apr-Sep
1d 7d 1d 7d
1d 7d 1d 7d
Aβ-crystalin, replicate 9
PM
LD
July-Jan
Apr-Sep
Ref
July-Jan
Apr-Sep
1d 7d 1d 7d
1d 7d 1d 7d
Aβ-crystalin, replicate 10

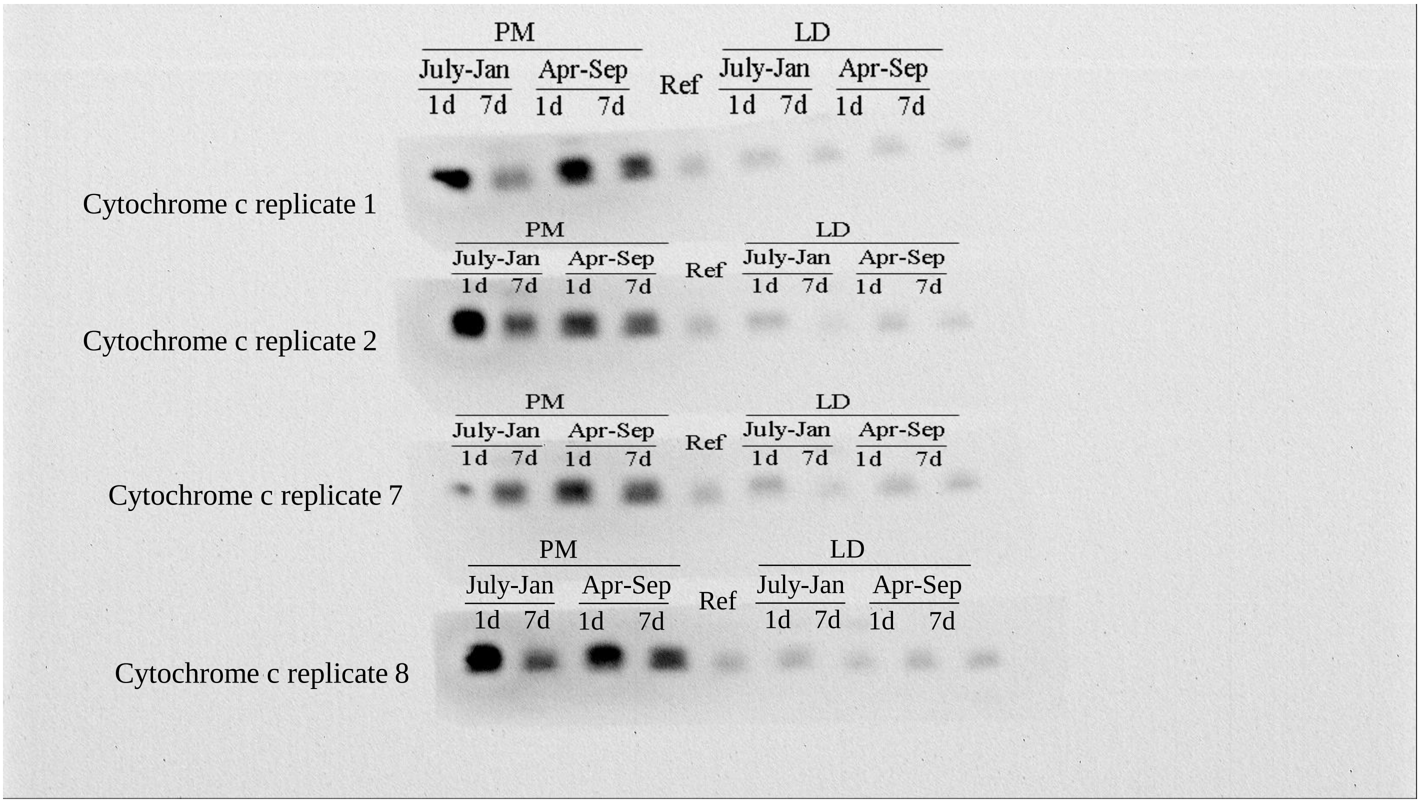
PM
LD
July-Jan
Apr-Sep
Ref
1d
7d
Cytochrome c replicate 1
Cytochrome c replicate 2
Cytochrome c replicate 7
Cytochrome c replicate 8

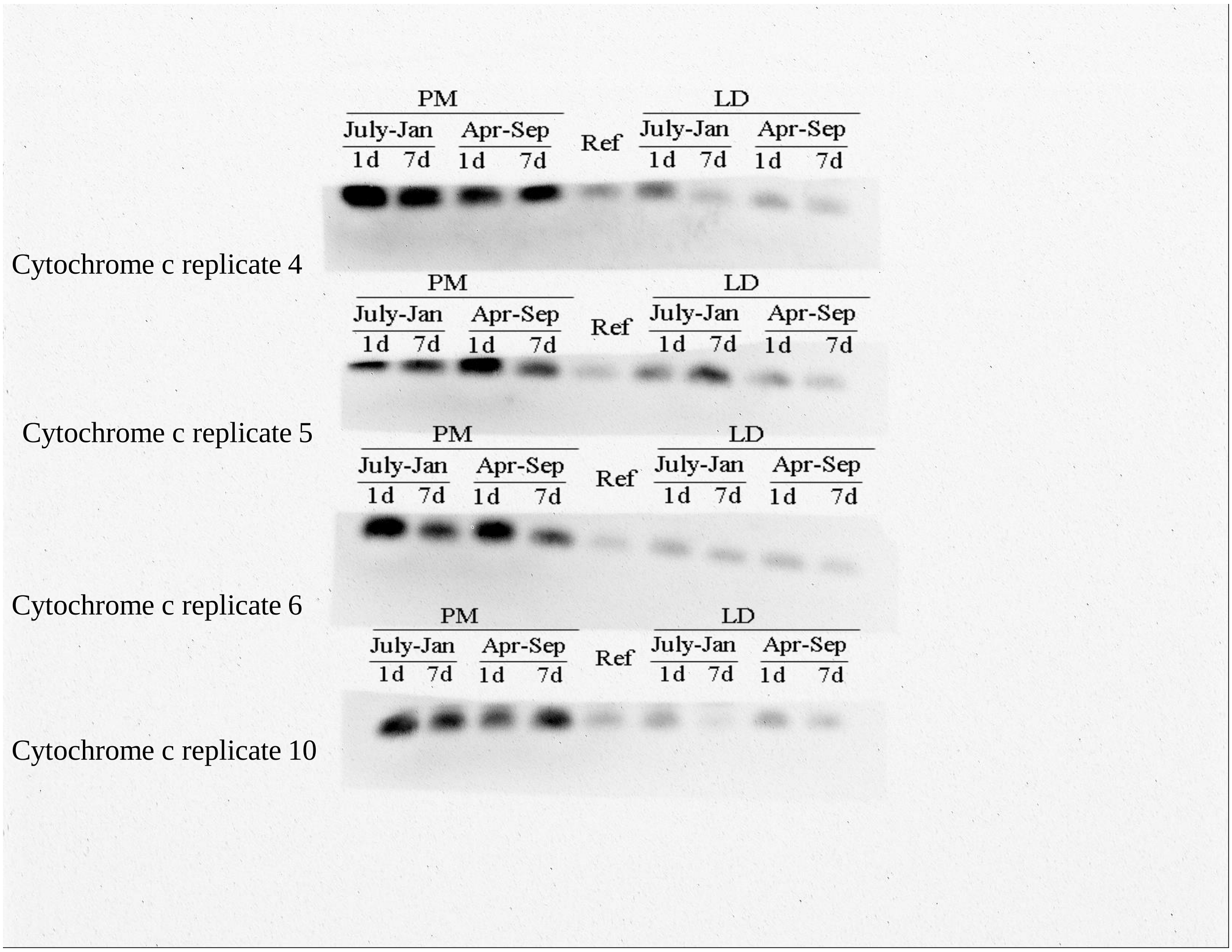
PM
LD
July-Jan
Apr-Sep
Ref
July-Jan
Apr-Sep
1d 7d 1d 7d
1d 7d 1d 7d
Cytochrome c replicate 4
Cytochrome c replicate 5
Cytochrome c replicate 6
Cytochrome c replicate 10
